# Magnetic Hyperthermia in Glioblastoma Multiforme Treatment

**DOI:** 10.3390/ijms251810065

**Published:** 2024-09-19

**Authors:** Veronica Manescu (Paltanea), Iulian Antoniac, Gheorghe Paltanea, Iosif Vasile Nemoianu, Aurel George Mohan, Aurora Antoniac, Julietta V. Rau, Stefan Alexandru Laptoiu, Petruta Mihai, Horia Gavrila, Abdel Rahim Al-Moushaly, Alin Danut Bodog

**Affiliations:** 1 Faculty of Material Science and Engineering, National University of Science and Technology Politehnica Bucharest, 313 Splaiul Independentei, District 6, RO-060042 Bucharest, Romania; veronica.paltanea@upb.ro (V.M.); antoniac.iulian@gmail.com (I.A.); antoniac.aurora@gmail.com (A.A.);; 2 Faculty of Electrical Engineering, National University of Science and Technology Politehnica Bucharest, 313 Splaiul Independentei, District 6, RO-060042 Bucharest, Romania; iosif.nemoianu@upb.ro (I.V.N.);; 3 Academy of Romanian Scientists, 54 Splaiul Independentei, RO-050094 Bucharest, Romania; 4 Faculty of Medicine and Pharmacy, University of Oradea, 10 P-ta 1 December Street, RO-410073 Oradea, Romania; 5 Department of Neurosurgery, Clinical Emergency Hospital Oradea, 65 Gheorghe Doja Street, RO-410169 Oradea, Romania; 6 Istituto di Struttura della Materia, Consiglio Nazionale delle Ricerche (ISM-CNR), Via del Fosso del Cavaliere 100, 00133 Rome, Italy; giulietta.rau@ism.cnr.it; 7 Institute of Pharmacy, Department of Analytical, Physical and Colloid Chemistry, I.M. Sechenov First Moscow State Medical University, Trubetskaya St. 8, Build.2, 119048 Moscow, Russia; 8 Faculty of Entrepreneurship, Business Engineering and Management, National University of Science and Technology Politehnica Bucharest, 313 Splaiul Independentei, District 6, RO-060042 Bucharest, Romania; petruta.mihai@upb.ro; 9 Technical Sciences Academy of Romania, 26 Bulevardul Dacia, RO-030167 Bucharest, Romania; 10 Sfanta Maria Medical Center Ghencea, 43B Bulevardul Ghencea, RO-061696 Bucharest, Romania

**Keywords:** magnetic hyperthermia, magnetic nanoparticles, glioblastoma, oncology, combinatorial therapy

## Abstract

Glioblastoma multiforme (GBM) represents one of the most critical oncological diseases in neurological practice, being considered highly aggressive with a dismal prognosis. At a worldwide level, new therapeutic methods are continuously being researched. Magnetic hyperthermia (MHT) has been investigated for more than 30 years as a solution used as a single therapy or combined with others for glioma tumor assessment in preclinical and clinical studies. It is based on magnetic nanoparticles (MNPs) that are injected into the tumor, and, under the effect of an external alternating magnetic field, they produce heat with temperatures higher than 42 °C, which determines cancer cell death. It is well known that iron oxide nanoparticles have received FDA approval for anemia treatment and to be used as contrast substances in the medical imagining domain. Today, energetic, efficient MNPs are developed that are especially dedicated to MHT treatments. In this review, the subject’s importance will be emphasized by specifying the number of patients with cancer worldwide, presenting the main features of GBM, and detailing the physical theory accompanying the MHT treatment. Then, synthesis routes for thermally efficient MNP manufacturing, strategies adopted in practice for increasing MHT heat performance, and significant in vitro and in vivo studies are presented. This review paper also includes combined cancer therapies, the main reasons for using these approaches with MHT, and important clinical studies on human subjects found in the literature. This review ends by describing the most critical challenges associated with MHT and future perspectives. It is concluded that MHT can be successfully and regularly applied as a treatment for GBM if specific improvements are made.

## 1. Introduction

**Figure 1:**
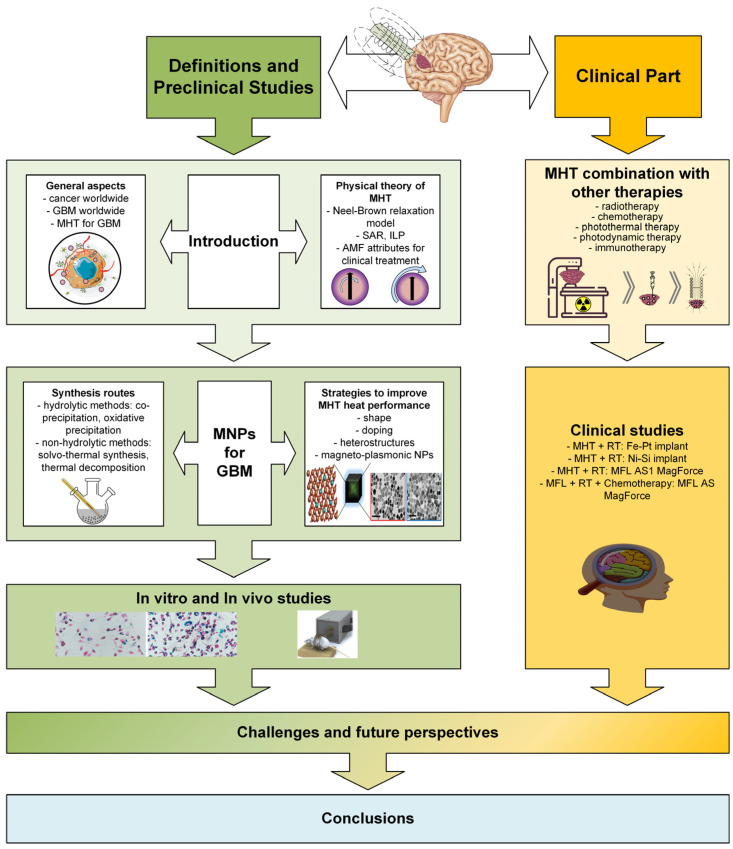
Flow sheet diagram depicting a summary of this study.

This review paper will briefly present worldwide cancer statistics to show the importance of the chosen subject, focusing on glioblastoma. Then, it will provide a physical description of magnetic hyperthermia with a clear explanation of magnetism and electromagnetic field phenomena. After that, the main synthesis routes for magnetic nanoparticles will be detailed with theory and examples extracted from the literature, and some strategies adopted to improve the specific absorption rate will be enumerated. In vitro and in vivo studies that consider the glioma cell lines of murine or human provenience and animal testing are provided, and some already applicable and possible combined therapies are presented with clear examples from the literature. This paper ends with clinical studies conducted over time that considered human trials involving magnetic hyperthermia in conjunction with other therapies and showed their positive and negative outcomes. Some challenges and future perspectives are underlined based on studies in the literature and the authors’ remarks. Figure 1 presents a flow sheet diagram containing a summary of the study with two main parts. The first one consists of definitions and preclinical studies and includes the paragraphs from Section 1, Section 2 and Section 3, while the second part, exhibiting a clinical part, includes Section 4 and Section 5.

### 1.1. General Aspects

Cancer is considered today to be associated with important health concerns, being the second disease in the world that leads to patient death [1]. For example, Bray et al. [2] found that about 9.6 million cancer deaths occurred worldwide in 2018. In addition, Siegel et al. [3] said in their study that about 1.8 million new oncological cases appeared and were diagnosed in 2020 in the United States of America (USA). Also, in a recent statistical analysis [4], it was established that 2 million new cancer cases and 611,720 cancer deaths will occur in 2024 in the USA. Increased incidences of cancer were noticed between 2015 and 2019, with an annual prevalence of 0.6–1% for cervical, breast, and pancreatic cancer and 2–3% for liver, kidney, prostate, and oral cancer. In Europe, it was estimated in the year 2020 that a number of 1.9 million new oncological diseases occurred in the case of women and about 2.1 million in men [5]. Regarding the African continent, the World Health Organization (WHO) estimated that cancer deaths will reach a value higher than 1 million per year by 2030. In 2022, 882,882 new cancer cases were accounted for, followed by 573,653 deaths [6]. It can be foreseen that the cancer death number will still be high in the future, and new treatments and efficient therapies will be continuously investigated and considered with the highest priority.

Cancer can be defined as an abnormal state in which healthy cells change their deoxyribonucleic acid (DNA) and multiply in an unregulated manner so that tumors with modified tissue properties occur. The cells inside these new tissue structures suffer cell mutation and exhibit different behavior compared to other healthy cells. In clinical practice, two types of tumors are distinguished: benign and malignant. Usually, benign cells are cataloged as non-dangerous because they only grow and develop in a localized manner without causing important damage to the healthy tissue placed in the tumor’s vicinity. On the other hand, malignant cells are the most invasive because, once they appear in a certain part of the human body, they enter the bloodstream and can populate different organs or structures, forming so-called metastases. These secondary growths are, in most cases, very aggressive and can have the potential to be life threatening [7]. Malignant tissue is characterized by a decreased pH value, high interstitial fluid pressure, decreased oxygen intake (hypoxia), chaotic blood vessels, which feed the tumor and maintain its viability, and deposits of dense stroma [8,9].

Glioblastoma multiforme (GBM) is frequently seen as a primary invasive brain tumor in the adult population and is, in most cases, associated with very poor prognoses and life expectancy. The standard surgical approach consisting of tumor resection and then administration of chemotherapeutical drugs or radiotherapy sessions is usually applied, but the estimated 5-year survival rate is seldom surpassed [10,11,12,13]. First-line treatment involves Temozolomide (TMZ) drug administration, but other drugs such as the monoclonal antibody Bevacizumab or combinations of lomustine, procarbazine, and vincristine (PCV) are approved and, in some cases, lead to cancerous cell death [10]. Almost always, patient outcome is reduced due to the fact that the tumor has no sharp edges, which does not permit its complete resection, and the risk of supplementary brain damage must be assumed. In addition, GBM can have different transcriptional profiles, such as classical, proneuronal, neuronal, and mesenchymal [14,15,16], and each of them is associated with different treatment approaches and life expectancies. In the USA, the average annual age-related rate of GBM apparition is equal to 3.21 per 100,000 people based on statistical analysis performed between 2011 and 2015 [17]. The median age of disease onset was established to be 65 years and mainly occurred in the case of men. The highest incidence was noticed in the case of non-Hispanic white populations, with an average annual age-adjusted incidence rate per 100,000 people of 4.71, 1-year relative survival of 41.4%, and 5-year relative survival of 5.4%. In addition, GBM occurrence is high in Australia, North America, and Europe [18]. In the USA, an overall prevalence of 9.23 per 100,000 people was reported [17]. From the risk factors associated with this type of cerebral cancer, one can mention exposure to ionizing radiations [19], rare genetic syndromes (Li–Fraumeni syndrome and Lynch syndrome), and increased patient age [10].

Recently, nanomaterials were introduced as a treatment in medicine, generating a new area called nanomedicine [20,21,22,23,24,25]. Many studies in this field were developed during this time, starting with clinical approval of different nanoparticles used in diagnosis and targeted therapy for different types of oncological tumors [26]. One of the most used strategies is hyperthermia, which consists of increasing the temperature above 40–43 °C in the affected tissue with the help of microwave, ultrasound, or radiofrequency waves, as well as based on laser tools or magnetic nanoparticles (MNPs) [27,28,29]. In [30], Fe_3_O_4_ magnetic nanoparticles were manufactured with a SiO_2_ shell and then loaded with TMZ. They were then coated with a platelet membrane to increase the biocompatibility of the nano assembly and improve drug distribution across the blood–brain barrier (BBB). The system was developed to act as synergistic therapy dedicated to GBM treatment, including the possibility of using MRI combined with photothermal and chemotherapy strategies. This approach proved to have high efficiency in inducing cellular death in an in vitro study performed on U251 and U87 malignant cell lines. Enhanced pH-responsive drug release combined with a photothermal effect were noticed. A temperature increase of about 65 °C led to the death of 80% of GBM cells.

**Figure 2:**
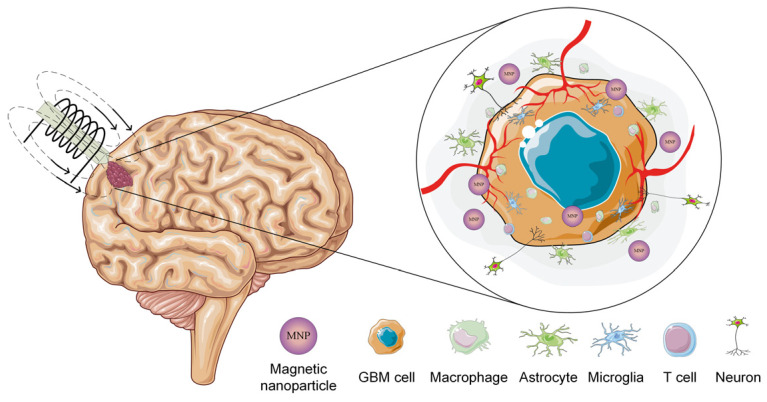
MHT used for GBM treatment and the GBM tumor heterogeneous microenvironment. This Figure presents how GBM cells recruit the microglia, macrophage, astrocyte, and T cells to enhance angiogenesis and tumor development while reducing the inflammation at the tumor site. Supplementarily, a hypoxic medium occurs inside the tumor, favoring the cancer cells localized at the tumor edge to invade the healthy tissue. This Figure was generated using images assembled from Servier Medical Art, which are licensed under a Creative Commons Attribution 3.0 unported license (https://smart.servier.com, accessed on 27 June 2024).

In addition, it is well known that MNPs, when subjected to the action of an alternating magnetic field (AMF), generate heat due to a transformation from magnetic energy to thermal energy. The potential of magnetic hyperthermia (MHT) was investigated in a direct relationship with the GBM treatment because, as stated before, these types of tumors are highly invasive and, in most cases, are resistant to classical approaches such as chemotherapy and radiotherapy due to the selectivity of the BBB, which permits the passage of lipophilic and small proteins without the necessity of special ionic channels. So, as a direct consequence, cerebral cancers are hard to destroy [13]. The treatment of GBM based on MHT is characterized by the administration of MNPs in the cancerous tumor, and then an AMF is applied (Figure 2). It results in an increase in tissue temperature between 41 and 43 °C as a direct consequence of the magnetic induction (*B*) value and the frequency (*f*) of the applied magnetic field. Different studies in the literature have investigated these treatments’ in vitro and in vivo efficiency and have shown that they have a high potential to increase patient life expectancy without the collateral effects of classical approaches [31,32].

### 1.2. Physical Theory of Magnetic Hyperthermia

To correctly understand and apply MHT in treating GBM, it is of utmost importance to briefly analyze the physical theory behind this phenomenon. MHT is an electromagnetic energy conversion to heat due to an interaction between MNPs and an external AMF [33]. When a time-variable magnetic field is applied, the MNP magnetic moments align parallel to the field direction. In some cases, minor deviations can occur as a function of material attributes such as chemical composition; crystallinity grade, size, and shape; the maximum value of magnetic field strength (*H*); and temperature (*T*). It is well known that under the action of a uniform increasing *H*, an MNP response can be quantified in a variable physical quantity called magnetization (*M*) [34]. The dependence, *M*(*H*), represents the hysteresis cycle of the magnetic particle and provides us with parameters such as coercivity (*H*_c_), saturation magnetization (*M*_s_), and remanent magnetization (*M*_r_). Generally speaking, the magnetic behavior of a bulk magnetic material is governed by the existence of magnetic domains separated by domain walls [34]. In the case of nanoparticles below a given diameter, the single-domain state is much more favorable from an energetic point of view than the multi-domain one [35]. This critical size is obtained from a balance equation between different types of energies. The magnetic moment of a single-domain particle exhibits two stable antiparallel orientations along the easy magnetization axis of the MNP. To switch from one state to another, one must pass an energy barrier, namely anisotropy energy, which depends on the particle volume (*V*) and the first anisotropy constant (*K*). The height of the barrier energy level is directly proportional to the particle size [36]. The thermal energy is computed as *k*_B_*T*, where *k*_B_ is the Boltzmann constant, and *T* is the temperature. When this type of energy has high values, the MNP spins will be located randomly along one of the two stable orientations, so, as a direct consequence, the total magnetization has an average value equal to zero. This particular behavior is called superparamagnetism, and coercivity is null in this case. When applying an external magnetic field, superparamagnetic particles have high magnetic saturation values concomitant with a null value of the remanence. It can be observed that superparamagnetic MNPs do not exhibit a hysteresis cycle, just a non-linear *M*(*H*) dependence. In MHT treatments, materials with superparamagnetic behavior at the body temperature of 37 °C are usually chosen because, in the absence of an AMF, they are inert and do not heat themselves. On the other hand, they are able to be rapidly controlled by applying a magnetic field. Some important aspects worth mentioning are as follows: the critical volume (*V*_c_) of the MNPs is considered to be a logarithmic function of time (*t*) (*KV*_c_= ln(*tf*_0_), where *f*_0_ is about 10^9^ s^−1^), and it can be easily noticed that in the low-frequency domain, the particles are in a superparamagnetic state, but in the higher frequency range the hysteresis cycle becomes prevalent [37]. When an AMF is applied, the MNPs have a hysteresis cycle, and the heat generated by a particle is given by the hysteresis cycle area (*W* [J/m^3^]), known under the name of specific hysteresis losses (Equation (1)).

(1) $$W=\int \mu_{0}M\left(H\right)HdH,$$

where $\mu_{0}=4\pi 10^{-7}[H/m]$ is vacuum magnetic permeability. In order to compute the specific heat dissipation power *P* [W/m^3^], one can use Equation (2).

(2) P=Wf,

where *f* represents the frequency of the applied AMF. The Rosenweig model [38] is usually applied and provides a relationship between the MNP attributes and hysteresis cycle area. In the framework of the MNP dynamic behavior, the model takes into account linear dependence between the magnetization vector and magnetic field strength vector, according to Equation (3).

(3) M=χH,

where χ is magnetic susceptibility. Considering the model in the complex number domain, one can assume that $\chi =\chi^{\prime }-i\chi^{\prime \prime },$ in which *i* denotes the complex part of the number and χ″ is associated with the part of the magnetization vector that is not in phase with the applied magnetic field. It is usually called the loss component of magnetic susceptibility. This particular case of a linear response (LRT) was developed into a Neel–Brown relaxation model [36] that is perfectly applicable in the case of superparamagnetic MNPs, low magnetic field strength, and magnetic susceptibility independent of magnetic field intensity. In [38], it was shown that, in this case, specific heat dissipation power could be computed as a function of the loss component of the susceptibility (Equations (4) and (5)).

(4) $$P=\mu_{0}\pi H_{\max}^{2}f\chi^{\prime \prime },$$

(5) $$\chi^{\prime \prime }=\chi_{0}\frac{\omega \tau }{{\left(1+\omega \tau \right)}^{2}},$$

where *H*_max_ is the maximum field strength, $\chi_{0}=\frac{M_{s}^{2}V}{3k_{B}T}$ represents static susceptibility, τ [s] is the relaxation time, and ω=2πf is the magnetic field angular frequency. The relaxation time can be expressed as a sum of two components (τ*_N_*—Neel relaxation time related to thermal variations in the MNP magnetic moments, τ*_B_*—Brown relaxation time linked to the rotational fluctuations of the particle), according to [39], as follows:

(6) $$\frac{1}{\tau }=\frac{1}{\tau_{N}}+\frac{1}{\tau_{B}},\tau_{N}=\tau_{0}e^{\frac{KV}{k_{B}T}},\tau_{B}=\frac{3V_{H}\eta }{k_{B}T},$$

where τ_0_ is between 10^−13^ and 10^−9^ s [40], *V*_H_ is the MNP hydrodynamic volume, and η is the viscosity of the magnetic fluid in which MNPs are immersed.

In the case of MHT treatments, specific power loss, namely the specific absorption rate (SAR) also known as specific loss power (SLP) or specific power absorption (SPA), must be analyzed. It is defined as the heat rate value at which the AMF absorbs electromagnetic energy and converts it into heat. It can be easily computed when the density (*d*) of the MNPs is known, according to Equation (7) [41].

(7) $$SAR=SLP=SPA=\frac{P}{d}.$$

**Figure 3:**
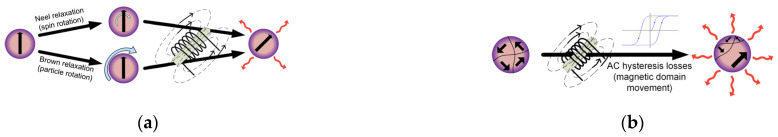
Schematical representation of heat release mechanisms under AMF effect in (**a**) single-domain MNPs and (**b**) multi-domain MNPs.

The linear response theory becomes inadequate for the MNPs situated near the transition between the superparamagnetic and ferromagnetic states, and the Stoner–Wohlfarth (SW) model must be applied [42]. In the case of clinical applications based on MHT, this phenomenon occurs only seldomly near the transition point between single-domain and multi-domain states (Figure 3). Some general approaches [37,43], also provide a combination of LRT and SW theories, but many researchers consider the LRT model to accurately describe the superparamagnetic behavior of most of the MNPs used in MHT treatments. In some cases, it is preferred to provide a normalized value of the SAR, called intrinsic loss power (ILP), with magnetic field characteristics (Equation (8)).

(8) $$ILP=\frac{SAR}{H^{2}f}.$$

Equation (8) is applicable only to frequencies in the MHz range, magnetic field strengths lower than the saturation field, and superparamagnetic particles.

One can immediately notice from Equation (4) that the heat dissipation power depends on the AMF attributes, such as the quadratic value of the maximum magnetic field strength and field frequency. It is expected when using MHT to treat oncological disease to establish given values for the product *H* × *f* [A/(ms)]. From the Maxwell–Hertz theory, it can be established that the eddy current and specific heat are directly proportional to (*H* × *f*)^2^.

In a study developed by Mamiya et al. [44] based on a model of the human body, the above-mentioned product was established to be about 2 × 10^9^ A/(ms). Hergt et al. [45] mentioned that the *H* × *f* values must be lower than 5 × 10^9^ A/(ms). Related only to cancers, Thiesen and Jordan [46] reported a value of 4.2 × 10^9^ A/(ms) for GBM treatment and 1.6 × 10^9^ A/(ms) in the case of prostate carcinoma. At the present moment, there is no standard limit for the value of *H* × *f*, but it is overall accepted that it must be chosen in good accordance with medical application, and its value must be established by considering that the body tissue overheating effect due to induced eddy currents has to be avoided. Generally, there are accepted values below the superior limit of 5 × 10^9^ A/(ms), and each research group should perform in vivo experiments to see the results. Today, most MHT devices approved for human use can generate an AMF with a frequency of 0.05 ÷ 1.2 MHz and an amplitude of a maximum of 5000 A/m. On the other hand, in clinical trials for GBM treatment, a magnetic field characterized by a frequency of 100 kHz and an amplitude of 18 kA/m was applied to brain tumors in multiple treatment sessions [47].

## 2. Magnetic Nanoparticles Used in MHT Treatment for GBM

To enumerate the main magnetic requirements for MNPs, one must underline the fact that they need a high value of *M*_s_ because this will involve an important amount of thermal energy dissipation in tumor cells, as well as increased control of MNP movements in the blood when an external magnetic field is applied. The MNPs have to be in a superparamagnetic state; this means that their coercivity and remanence should be very low because, in the absence of an AMF, they exhibit any magnetic properties at temperatures higher than the blocking temperature, and, in this way, aggregations of particles are prohibited, and colloidal stability is maintained. The size of the particles must be in the nanometer range to generate low dipolar interactions between them [48].

It can be foreseen that MNP attributes such as saturation magnetization, remanent magnetization, coercivity, first anisotropy constant, particle diameter, and shape are very important in establishing a proper value for SAR [48]. From the literature, it was noticed that *M*_s_ values were lower than 200 emu/g [49,50], an appropriate diameter of particle *D* was chosen between 10 and 40 nm correlated with a volume *V* of 0.5 ÷27 × 10^3^ nm^3^, and the first anisotropy constant was between 8 × 10^2^ J/m^3^ and 1.6 × 10^2^ J/m^3^ [36]. Regarding coercivity, values between 170 and 400 Oe were reported in the case of Fe_3_O_4_ MNPs with a diameter of about 17 nm and a spherical shape [51,52]. In most cases, the ratio of *M*_r_/*M*_s_ was between 0.1 and 0.7 [36]. Magnetic hyperthermia in humans is usually performed based on iron oxide nanoparticles (IONs) because they have an increased heat capacity [53]. The main iron oxides used in nanomedicine are magnetite Fe_3_O_4_ and maghemite Fe_2_O_3_ [35].

Table 1 provides the magnetic and structural properties of MNPs used in MHT experiments.

As can be observed from Table 1, preparation methods and particle features are of utmost importance in obtaining optimal heat performance. It can be concluded that particle crystallinity grade, size, and size distribution must always be analyzed. In addition, chemical composition, magnetic properties, and anisotropy can have an effect on magnetic cluster apparitions if the medical application requires them. Otherwise, they can dictate to particles to remain independent.

### 2.1. Synthesis Routes for MNP Manufacture Used in MHT

**Figure 4:**
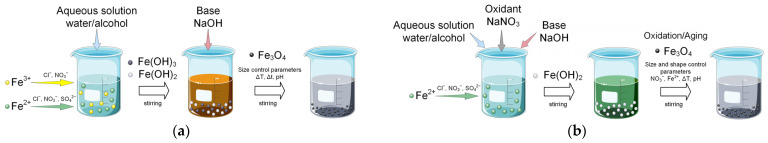

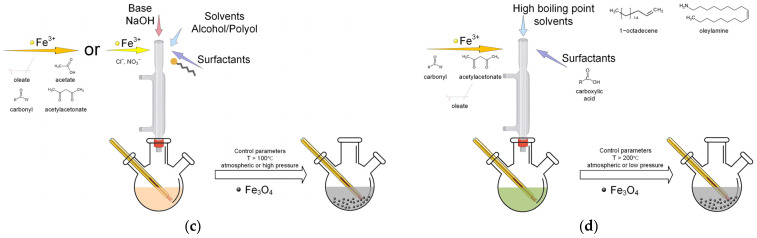
Synthesis routes for MNP manufacture based on sol–gel approaches. Hydrolytic methods: (**a**) chemical co-precipitation; (**b**) oxidative precipitation. Non-hydrolytic methods: (**c**) solvothermal/polyol synthesis; (**d**) temperature thermal decomposition. This Figure was generated using images assembled from Servier Medical Art, which are licensed under a Creative Commons Attribution 3.0 unported license (https://smart.servier.com, accessed on 27 June 2024).

Usually, MNPs for MHT applications use wet chemistry technology, which is defined as a chemical process conducted in a liquid medium [66]. Frequently, sol–gel processes are involved, and they consist of an inorganic colloidal suspension (sol) apparition, followed by sol gelation and its transformation into a continuous liquid phase known as a gel [36]. Taking into account the liquid in which MNPs are introduced, one can mention the hydrolytic and non-hydrolytic sol–gel processes that will be described in this section (Figure 4).

When it comes to preparing magnetite or maghemite nanoparticles, hydrolytic methods stand out as the techniques of choice. The most prevalent one is the co-precipitation of two metal salts, M^2+^ and M^3+^, and the oxidative precipitation of M^2+^ salts [67]. This synthesis route typically starts with the dissolution of metal salts into a solution obtained through a combination of water and alcohol. The addition of a strong base in some cases can lead to the formation of insoluble species such as Fe(OH)_2_ and Fe(OH)_3_, which then transform into magnetite (Fe_3_O_4_). The following chemical reaction illustrates this process:

(9) $$2{\mathrm{Fe}}^{3+}{+\mathrm{Fe}}^{2+}\to {2\mathrm{Fe}(\mathrm{OH})}_{3}{+\mathrm{Fe}(\mathrm{OH})}_{2}\to {\mathrm{Fe}}_{3}O_{4}{+4H}_{2}O.$$

One of the most important steps in this process consists of water deprotonation generated by cation solvation, hydrolysis that determines the apparition of hydroxide complexes M-OH, condensation, which is linked to M-O-M polymeric framework apparition, and finally, generating the M_x_Fe_3-_xO_4_ compound [68]. The MNPs’ sizes can be easily tuned by changing the time, temperature, and pH. In this direction, Nkurikiymfura et al. [69] synthesized, through the co-precipitation method, Fe_3_O_4_ magnetic nanoparticles with a size of 11.22 nm at room temperature under a stirring rate of 200 rpm and a pH value of 10. This outcome is special because the literature had reported that superparamagnetic MNPs with a reduced particle size were usually produced at about 80 °C concomitantly, with a high stirring rate between 300 and 1500 rpm and/or under ultrasound waveforms. Mascolo et al. [70] used the same co-precipitation method for MNP synthesis at room temperatures and a large range of pHs based on different strong bases such as NaOH, (C_2_H_5_)_4_NOH, and KOH. They noticed that the pH value, chemical composition of the basic solution, and the rate of basic solution addition to the bivalent or trivalent iron highly influenced the MNPs’ sizes. Karaagac et al. [71] investigated the effect of the stirring rate and NaOH concentration on the MNPs’ size and magnetic properties. They noticed that MNPs with an average size of 7.4 nm were obtained at a stirring rate of 1100 rpm. These particles exhibited an average magnetic saturation of about 70.4 emu/g. In addition, the authors noticed that the saturation magnetization value can be controlled based on NaOH concentration, which must be higher than 5.5, and the stirring velocity.

Regarding the other hydrolytic route, namely oxidative precipitation, a partial oxidation reaction of Fe^2+^/M^2+^ salts, with M being a transition metal, occurs in an alkaline media such as NaOH under the action of an oxidative substance (NaNO_3_) [36]. An intermediate phase is formed due to base presence, which suffers a dihydroxylation process followed by magnetite apparition. The chemical reactions from Equation 10 characterize this synthesis route.

(10) $$\begin{array}{l} {3\mathrm{FeCl}}_{2}+6\mathrm{NaOH}\to {3\mathrm{Fe}(\mathrm{OH})}_{2}+6\mathrm{NaCl},\\ {2\mathrm{Fe}(\mathrm{OH})}_{2}+0.5O_{2}\to 2{\mathrm{FeOOH}+H}_{2}O,\\ {2\mathrm{FeOOH}+\mathrm{Fe}(\mathrm{OH})}_{2}\to {\mathrm{Fe}}_{3}O_{4}{+2H}_{2}O. \end{array}$$

Marciello et al. [72] proposed an aqueous synthesis of up to 20 g of MNPs with a size between 20 and 30 nm close to the superparamagnetic–ferrimagnetic limit. For particles with a size bigger than 35 nm, the saturation magnetic moment was about 90 Am^2^/kg, and the coercive force (μ_0_*H*_C_) was estimated at 10 mT, while in the case of smaller MNPs with a size of 22 nm, a reduction in saturation magnetic moment (82 Am^2^/kg) and coercive force (3 mT) were observed. These values were very close to those of the superparamagnetic limit. SAR measurements were made by considering the following test conditions—70 kHz/44 mT and 102 kHz/20 mT—as it was already established that, in biomedical applications, these values must be in the 50–1200 kHz frequency range and in the 0–20 mT magnetic induction range, as presented in Section 1. The SAR values were between 95 and 170 W/g. Verges et al. [73] proposed a direct method for the preparation of Fe_3_O_4_ MNPs with a size of 30 nm and stability in aqueous media at a pH of 7. They used Fe^2+^ salt (FeSO_4_) under the effect of NaOH and KNO_3_. They found values for *M*_s_ between 83 and 92 emu/g and for *H*_c_ between 50 and 100 Oe. These values were very close to those at which the coherent mechanisms of magnetization rotations occurred in good accordance with the Stoner–Wohlfarth model [34]. The authors also performed MHT measurements based on calorimetric experiments and found that the maximum SAR value of 95 W/g was obtained for colloidal suspension with a concentration of 5 mg/mL. This value was appropriate for GBM treatment based on MHT, being one of the highest reported in the literature. Based on the classical oxidative precipitation method, Antoniac et al. [74] prepared a mixture of MNPs, γ–Fe_2_O_3_, α–Fe_2_O_3_, and Fe_3_O_4_, which were coated with biocompatible polymers. The particles had sizes between 40 nm and 118 nm and Zeta potentials between −29.7 mV and −62.7 mV. When introduced into a hyaluronic acid-based solution, no agglomerations of MNPs were observed. The authors concluded that by coating the size of the particle, it can be reduced concomitantly with an increase in biocompatibility.

Usually, after hydrolytic methods are performed, some special steps are required to enhance particle monodispersity and lower the standard deviation of the size, such as microfiltration, size-sorting, static magnetic fractionation, or ultracentrifugation [75,76,77]. These operations are important because a study developed by Bae et al. [78] proved that, by sorting the MNPs, higher SAR values and improved MHT effects can be achieved. The authors found that chitosan oligosaccharide-stabilized ferrimagnetic iron oxide nanocubes with a size of about 30 nm exhibited a SAR of 2614 W/g compared to the commercial superparamagnetic particles Feridex^®^ (83 W/g).

Non-hydrolytic methods for MNP synthesis are based on alkyl oxygen derivates’ use as oxygen donors. These derivates react with Fe and generate its oxides, acting as a solvent and/or surfactant for a chemical reaction to stabilize the species that contain Fe inside the solution. Two main routes are found in the literature: solvothermal methods and organic precursor thermal decomposition. The solvothermal synthesis uses solvents such as polyols (ethylene glycol and diethylene glycol) or alcohols (1-octanol, ethanol, 1,2-hexanediol), two types of metal precursors (metal salts–ion chlorides or nitrates or metal-coordination complexes—iron–carbonyl, iron–oleates, and acetylacetonates/acetates), and surfactants such as water-soluble polymers, organic molecules, or surfactants with a hydrophobic tail as a function of the involved alcohol chemical formula [36]. Lin et al. [79] prepared based on solvothermal synthesis hollow Fe_3_O_4_ spheres. They used ferric chloride, ethylene glycol, urea, and ammonium acetate. A homogenous solution was made, dispersed, poured, and sealed into an autoclave for a maximum time interval of about 24 h up to 200 °C. Magnetite was present as ferric ions located on the hollow spheres. Another study performed by Tian et al. [80] synthesized 4–6 nm MNPs through this method based on Fe(acac)_3_, n-octylamine, n-octanol, solvents, and reducing agents. The authors concluded that, in this way, high-quality magnetic nanoparticles were produced without needing a high heat transfer value, as in the case of thermal decomposition. The research presented in [81] put into evidence the importance of the temperature at which the solvothermal method is performed. The authors reached a temperature below 140 °C, but the MNPs exhibited low saturation magnetization, which was inadequate for MHT treatments. It was concluded that the magnetic properties of the MNPs strongly depend on the particle crystallinity grade and the temperature at which the process is performed. There are a limited number of studies in the literature that discuss the MHT results for MNPs prepared through solvothermal methods. For example, Das et al. [82] prepared, based on a one-step solvothermal process, Ag/Fe_3_O_4_ nanoparticles at a standard temperature of 200 °C for 24 h. The MNPs exhibited a highly crystalline Ag monodomain of about 45 nm, and Fe_3_O_4_ randomly oriented crystallites around it. Magnetic measurements were carried out in DC based on a vibrating sample magnetometer, and the value of saturation magnetization was established to be between 62 emu/g and 90 emu/g. The MHT experiments were made in water and agar. The last medium was chosen to simulate the viscosity of cancer cells. A SAR value of about 160 W/kg was obtained for a magnetic field strength of 800 Oe. The authors concluded that the developed particles are a good candidate for MHT treatments. Hugounenq et al. [60] manufactured iron oxide monocrystalline nanoflowers using a modified “polyol” strategy. The particles were constituted of 11 nm grains, which formed a flower-like structure. The *M*_s_ was found to be about 69 emu/g, a value that is very close to that of bulk material, and an interesting compromise between a high SAR value of about 1992 W/kg and a size of 24 nm was noticed. However, besides the fact that the physical theory behind the thermal loss processes must be further investigated, the authors concluded that the developed nanoflowers exhibited proper characteristics to be applied in cancer treatment based on MHT. The solvothermal method exhibits an important advantage because the prepared MNPs could be easily transferred to water and directly applied to MHT. In addition, the method is characterized by some disadvantages such as a reduced quantity of MNPs in the order of milligrams, particle solubility depending on the high amount of surfactants that are used in the process, and the fact that in almost all the cases, supplementary processing steps are necessary. 

The thermal decomposition (TD) route includes the use of an organic solvent that has an increased boiling point and is inert at a temperature of about 200 °C, which permits metallic precursor decomposition. Usually, solvents such as benzyl ether, 1-octadecene, octyl ether, or benzyl ether and metal precursors such as carbonyls, acetylacetonates, or oleates are involved in the process [36]. The shape, size, and aggregation of the MNPs depend on the surfactant nature and the number of nuclei that appear in the first nucleation step. The most used surfactants in the thermal decomposition method are oleic acid, trioctylphosphine oxide, decanoic acid, oleyamine, or quaternary ammonium salts. To obtain a proper result from a chemical reaction, this is conducted at an atmospheric or reduced pressure condition. Gonzales-Weimuller et al. [83] based their design on the thermal decomposition of iron oxide nanoparticles with sizes between 5 and 14 nm. They used octyl ether, iron pentacarbonyl, trimethylamine N-oxide, oleic acid, and chloroform. The initial magnetic susceptibility had value in a 0.93 (5 nm diameter MNPs) ÷ 12.31 (14 nm diameter MNPs) interval, while the SAR measured at a frequency of 400 Hz, and the applied magnetic field strength of 24.5 kA/m was between 130 W/kg (10 nm diameter MNPs) and 447 W/kg (14 nm diameter MNPs). The authors demonstrated that the SAR value has an important variation as a function of particle size and that through the TD synthesis route, particles with different sizes and shapes adequate for MHT applications can be obtained. Salas et al. [84] prepared uniform iron oxide MNPs with different sizes (14–22 nm) through TD of an iron oleate complex in 1-octadecene by controlling the nucleation and growth processes. They chose, for SAR estimation, the frequency of an external AMF of 77 kHz, with amplitude for magnetic field strength of 39.78 kA/m, and found values between 70 and 95 W/g. Regarding the ILP, a variation between 0.33 and 0.78 nHm^2^/kg was achieved. It was concluded that faceted MNPs can be successfully used for oncological treatments. Guardia et al. [56] used iron(m) acetylacetonate, decanoic acid, dibenzyl ether, squalane, gallic acid, poly(ethylene glycol), N,N′-dicyclohexylcarboiimide, and 4-dimethylaminopyridine to prepare, based on a TD synthesis protocol, well-defined cubic particles with sizes between 14 and 35 nm and octahedral- or truncated-octahedral-shaped (sizes 40–100 nm) MNPs by adjusting the dibenzyl ether–squalane ration. The SAR value for cubic MNPs was established to be between 360 W/g (14 nm MNPs) and 650 W/g (24 nm MNPs), and in the case of octahedral 18 nm sized MNPs, the value was about 124 W/kg, while for truncated octahedra 22 nm sized MNPs, it was estimated to be around 95 W/kg. Also, this study evidenced that the TD method is adequate for obtaining different shapes and magnetic behaviors of MNPs as a function of the chemical reactive ratio used in the synthesis steps. Because the TD synthesis route is characterized by an increased temperature value and because the surfactant uses specific kinetics of the chemical reactions, the crystallinity grades, sizes, and shapes of the MNPs can be quickly achieved compared to hydrolytic routes. As mentioned before, the process is tunable and can be modified as a function of precursor choice, solvent, molecule stabilizer substances, and the presence/absence of a temperature ramp, as well as by adjusting the constituent concentration in solutions to adapt, as much as possible, the MNPs’ magnetic and thermal properties and to design various solutions for patient-adapted oncological strategies.

Table 2 presents examples of MNPs prepared based on hydrolytic and non-hydrolytic routes, with information regarding the synthesis method, particle size and shape, SAR values, and *H* × *f* product.

From the studies presented in Table 2, it can be concluded that the highest SAR values were obtained for the solvothermal synthesis route and nanoflower γ-Fe_2_O_3_ magnetic particles for an *H* × *f* product in biological limits [60], followed by thermal decomposition applied for spherical Fe_3_O_4_ MNPs, but in some cases beyond the biological range [83,89]. The co-precipitation method leads to lower SAR values compared to oxidative precipitation in the case of different types and mixtures of iron oxide nanoparticles [72,85,88]. This synthesis route is the most used in the industry to produce the already Food and Drug Administration (FDA)-approved MNPs such as Resovist^®^ and Feraheme^®^ [86,87]. Many synthesis routes and preparation methods are dedicated to MNPs, but they have not been extensively investigated in the literature for their direct relationship to the MHT phenomenon. In our opinion, for recurrent GBM tumor treatment, only one of the safest and known routes must be addressed, and much more research is needed to improve their magnetic properties and biocompatibility to be adequate not only for primary localized tumor treatment but also for small metastases, in which only a low quantity of MNPs can reach, and, to exhibit a destructive effect on cancer cells, their heat capacity should be higher. Considering BBB’s biological characteristics, only small MNPs coated with polymeric materials can pass without the necessity of ionic channels. Thus, we consider there to be a gap in worldwide research regarding the improvement of hydrolytic/non-hydrolytic methods to design optimized MNPs for GBM treatment and alleviation.

### 2.2. Strategies Adopted to Improve MHT Heat Performance

Some important strategies can improve and modify MHT heat characteristics, such as particle shape, chemical composition of the MNPs, and magnetic heterostructure design [36,91].

As presented in Table 2, one can notice that although spherical MNPs are the most used in preclinical studies, their SAR value is not the highest compared to other particle shape heat performances. The FDA-approved MNPs exhibit a spherical geometry because manufacture synthesis is much easier for this case, and a large quantity of this product can be delivered faster to consumers. 

To achieve a shape-controlled process for nanocrystals (e.g., cubic, octahedral, flower, or rod), certain surfactants or ligands must be added, and the metal precursor’s and other chemical substances’ molar ratios must be carefully checked and adjusted as a function of different parameters such as time, pressure, heating rate, or temperature ramp. Guardia et al. [56,57] proved that dibenzyl ether used as a solvent generated, through a decomposition phenomenon, sub-products that were linked to increased control of the cubic shape for MNPs. The authors developed Fe_3_O_4_ MNPs with a 25 nm average cube edge and high SAR values of up to 650 W/g measured for an *H* × *f* product of about 4.8 × 10^9^ A/(ms). Other studies [92,93] showed that, by introducing aromatic ethers that follow a radical decomposition during the thermolysis process, magnetic nanocubes can be obtained, while research developed by Lee et al. [94] considered the cubic shape of the MNPs to be in a direct relationship with the thermal decomposition of Fe^3+^ oleate due to the existence of certain ligands such as alkaline metal reagents and sodium oleate. Another particle shape analyzed in the literature is the octopod, which is considered to create supplementary symmetry that positively influences surface anisotropy energy and increases particle heat performance. In this direction, Nemati et al. [95] used oleic acid and polyamine as stabilizers and made Fe_3_O_4_ nano octopods. The authors reported, for the SAR, a value of 60 W/g at 4.9 × 10^9^ A/(ms), very similar to that obtained for Resovist^®^. Another shape linked to cubic is octahedral. As presented before, Salas et al. [89] manufactured octahedral and truncated octahedral MNPs with an average size of about 20 nm by enhancing the growth rate along the <100> direction in detriment to the <111> direction. They proved the apparition of spherical polyhedral-shaped MNPs prepared with oleic acid [84]. It is well known that non-hydrolytic routes, such as solvothermal methods, lead to nanoflower synthesis. Hugounenq et al. [60] modified the protocol developed by Caruntu et al. [96] by introducing, in the synthesis protocol in diethylene glycol, a mixture of FeCl_2_/FeCl_3_ annealed in N-Methyldiethanolamine and NaOH and obtaining ION nanoflowers. A high value of SAR of about 500 W/g for an AMF field condition of 4.40 × 10^9^ A/(ms) was achieved for the particles with a size of 21 nm. Modifying the synthesis route can produce particles of different shapes, and, for the same chemical composition, increased SAR values can be achieved. This strategy agrees with recurrent GBM treatment, characterized by the need for heat-efficiency MNP use.

Another important strategy to increase the MNP’s heat efficiency involves doping classical iron oxide with transition metals. Usually, metal ferrites have the following chemical formula, MFe_2_O_4_, in which M is a divalent transition metal, and are characterized by a spinel structure with face-centered cubic arrangements of the oxygen atoms, while iron (Fe^2+^) and the other metal (M^2+^) ions are positioned in octahedral or tetrahedral places. It can be foreseen that the SAR can be improved due to the fact that these transition metal atoms have a characteristic value of the magnetic moment due to the existence of vacancies in the MNP structure. All the synthesis methods that are presented in Section 2.1. are suitable for producing MNPs of advanced chemical composition. The most used elements are Zn, Mn, and Co. Other soft magnetic structures [50], which are not spinels, such as Fe nanoparticles (NPs) (α-Fe), Fe carbide NPs (ε’-Fe_2.2_C), or alloys such as Ni-Cu [97], Fe-Co, and Fe-Ni-Co [98] were proposed but exhibited reduced SAR values outside the biological limits of the MHT treatment. Some of the best heat performances were observed in cases of Co_0.7_Fe_2.3_O_4_ nanocubes [99], Mn_0.7_Fe_2.3_O_4_ nanoflowers [100], and Fe and Fe carbide nanospheres [50]. Bordet et al. [101] noticed that when the iron carbide NPs were exposed to air and then inserted into water, they could passivate, and a decrease in saturation magnetization was noticed concomitantly with the build of a 3 nm thick iron oxide shell. Firstly, the SAR value of 15 nm MNPs was measured at 700 W/g; then, it increased in the case of Fe_2.2_C at 3250 W/g, and, after iron carbide MNP oxidation to Fe_3_O_4_ on the shell, it reduced to 1000 W/kg at an *H* × *f* product of 3.7 × 10^9^ A/(ms) after 4 months of heat transfer in water through dopamine-based ligands. These SAR values are much higher than those found in the case of commercial MNPs. Sathya et al. [99] prepared cobalt ferrite NPs through a non-hydrolytic method and found, in the case of cubic particles with a size between 17 ÷ 19 nm, a SAR value of 800 ÷ 900 W/g in a field condition of 4.8 × 10^9^ A/(ms). Manganese ferrites are one of the most suitable MNPs used in MHT due to their excellent chemical stability, important magnetic properties, and reduced toxicity. Some medical studies [102,103,104,105,106,107], linked Mn toxicity with a potential neurotoxic effect related to Parkinson’s disease and other symptoms such as difficulty walking, facial and limb tremors, and impaired speech. Andrade et al. [108] synthesized, through the sol–gel method, Ca_0.2_Mn_0.8_Fe_2_O_4_ MNPs with a SAR value of about 36.3 W/g that determined a temperature increase with about 7 °C in only 120 s, proving to have high potential for MHT applications. Silveira-Alves et al. [109] also prepared MnFe_2_O_4_ nanoparticles with porphyrin coated with citrate, dimercaptosuccinicnate, and tripolyphosphate anions. Saturation magnetization of the MNPs mentioned above was between 44 emu/g and 50 emu/g. Another study [110] investigated MnFe_2_O_4_ modification with polyethylene glycol (PEG) loaded with glucose oxidase and reported a SAR value of about 296 W/g and a saturation magnetization of 75 emu/g. Casula et al. [111] synthesized Mn-doped nanoflowers with an average size of about 57 nm using the polyol method. The authors experimentally determined a maximum SAR value of about 350 W/g at 4.8 × 10^9^ A/(ms). It can be observed that the efficiency of MHT treatments based on manganese MNPs is highly dependent on Mn^2+^ substitution, MNP size, number of substitution sites, characteristics of the AMF such as frequency and magnetic field strength, and the administrated dose of MNPs due to neurological side effects [112,113,114].

Jang et al. [115] prepared Zn-doped ferrites (Zn_0.4_Mn_0.6_Fe_2_O_4_) with a SAR value of about 432 W/g in the case of *H* × *f* = 2.6 × 10^9^ A/(ms) and 15 nm diameter spherical MNPs. While iron oxide NPs are already used in clinical trials, Zn-ferrites were investigated in pre-clinical studies to analyze their toxicity and safety field limits [116]. The chosen concentration of 200 μg_metal_/mL was considered safe, but the temperature of 43 °C was surpassed. However, a delay in tumor development in murine animal models was observed. Regarding Co-dopped ferrites, an in vivo study developed by Balakrishnan et al. [117] took into account the fact that Co induced signs of toxicity at concentrations of about 200 μg_metal_/mL. Based on the plasma micro-arc oxidation (PMAO) procedure, the authors modified the surface of Co-ferrite nanocubes with an edge of 17 nm and injected the magnetic solution in a murine xenograft tumor model. It was noticed that the tumor was eliminated after one dose of 0.7 mg fluid injection and three MHT cycles applied at 110 kHz and 20 kA/m. Although gadolinium (Gd)-based contrast agents have been widely used and considered safe, some studies reported side effects associated with the development of nephrogenic systemic fibrosis [118], anaphylactic shock, or other acute reactions [119,120,121]. This lanthanide can accumulate in kidneys, bones, or even the patient’s brain without noticeable renal malfunction. The main toxicity was associated with Gd^3+^ dissociation from the chelated complexes due to the interaction with different compounds in the extracellular matrix. Jiang et al. [122] developed Gd_0.02_Fe_2.98_O_4_ MNPs for MHT with beneficial effects such as necrosis and damage of the blood vessels at the tumor site and diminishing of the hypoxic cells resistant to a radiation process. They combined radiotherapy and MHT strategies. A high SAR value correlated with an increased temperature of 45 °C was reported, and the authors concluded that the developed MNPs exhibited good thermal behavior and offered the possibility to be also used as radiotherapy agents. Avugadda et al. [123] synthesized multifunctional composite nanostructures based on Fe MNPs combined with Gd MNPs in a multilayer structure. These complexes were dedicated to a simultaneous imaging process and MHT. A SAR value of about 85 W/g_Fe_ was achieved. The authors investigated the magnetic structure biocompatibility on U87 glioblastoma cells and noticed that a maximum Gd dose of 125 μg/L was not toxic. The developed composite exhibited outstanding properties for tracking tumor microenvironment release with remote T_1_ guidance and magnetic hyperthermia therapy actuation. Considering the high potential of the Gd-based MNPs in MHT, one can foresee that this solution can be successfully applied in GBM treatment if only specific Gd doses are respected [124]. 

Other chemical formulations, such as iron-based NPs or ion carbide, must be further investigated because in vivo results are missing in the literature. As an overall conclusion, it can be observed that doped composition exhibits increased heat efficiency, but safety measures regarding metallic toxicity must be considered, and biocompatible coatings or surface modifications should be applied. 

The synthesis of heterostructures represents another strategy adopted in material science to increase MHT heat performance. This domain includes bi-magnetic core–shells such as MNPs combined with ferrite core–shell, Fe core and iron carbide or ferrite core–shell, Co ferrite core and Zn ferrite shell, etc. Usually, the core–shell bi-magnetic structures are made based on a so-called seed growth method that consists of pre-formed seed NPs, which are used as a core, while the shell has to be characterized by a similar lattice structure [51]. In [51,125], CoFe_2_O_4_@MnFe_2_O_4_ and Fe_3_O_4_@CoFe_2_O_4_ were synthesized with excellent SAR values of 2000 W/g and 2778 W/g at an *H* × *f* value of 1.84 × 10^10^ A/(ms) but outside the biological limit. Another study [126] changed the shape of the core–shell particles from spherical to cubic and measured a SAR of 10,600 W/g in the case of ZnFe_2_O_4_@CoFe_2_O_4_ MNPs. By analyzing the literature, one can conclude that soft magnetic cores made of iron oxide, zinc ferrite, and manganese ferrite and hard magnetic shells such as cobalt ferrite can be linked to increased values of SAR, even though the studies were not performed in the biological range for the *H* × *f* product. The MNP concentration in ferrofluids can be adjusted to meet the clinical requirements. Other highly biocompatible core–shell structures were FeO@Fe_3_O_4_ developed by Lak et al. [63] and characterized by increased SAR values of 13-fold compared to the FDA-approved commercial Resovist^®^. Meffre et al. [50] developed a core–shell structure in which the core was made of metallic Fe MNPs, and the shell was designed from iron carbide. In this case, a very high SAR value of 3250 W/g was achieved in the clinical range of the applied AMF (3.24 × 10^9^ A/(ms)). A possible explanation of increased SAR values characteristic of bi-magnetic core–shell MNPs is attributed to the exchange coupling energy that determines an increased coercivity of the particles.

**Figure 5:**
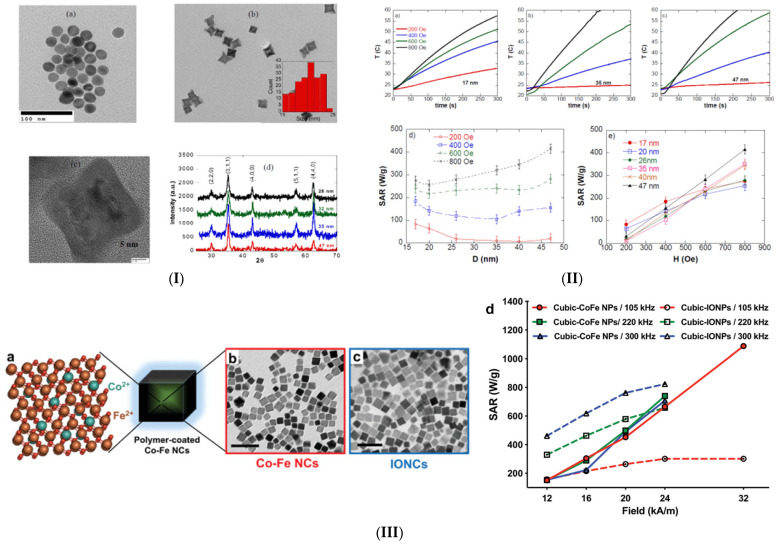

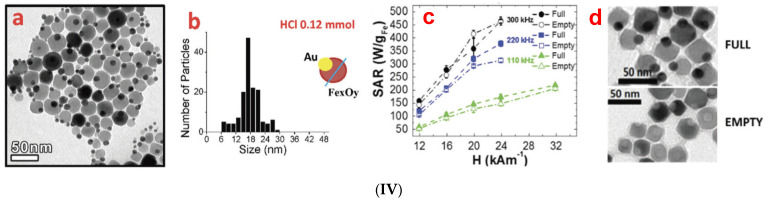
Strategies adopted to improve MHT heat performance. Shape and geometry influence on Fe_3_O_4_ MNPs: (**I**) transmission electron microscopy (TEM) images of (**a**) 25 nm spherical MNPs; (**b**) 20 nm nano-octopod MNPs; (**c**) high-resolution image obtained for 26 nm octopod particles; and (**d**) X-ray diffraction (XRD) spectra for different octopod particle sizes. (**II**) (**a**–**c**) calorimetric MHT curves for different particle sizes; (**d**) SAR variation as a function of particle size in the 200 ÷ 800 Oe field range; and (**e**) SAR values versus H values for various particle sizes. Reprinted with permission from [95]. Copyright (2024) American Chemical Society. Doping with transition metal influence: (**III**) comparison between Co-doped nano ferrite and classical iron oxide MNPs: (**a**) Schematical representation of magnetic aqueous solution; TEM images of (**b**) Co-doped nano ferrites; (**c**) classical iron oxide MNPs (scalebar 50 nm); and (**d**) SAR value variation obtained for different types of MNPs as a function of magnetic field strength at different frequencies of AMF [117]. Figure 5 (**III**) is licensed under CC BY 4.0. Manufacture of magneto-plasmonic NPs. (**IV**) Gold–iron oxide dimers for MHT applications: (**a**) Au-iron oxides dimer images; (**b**) statistical analysis performed to establish the average particle diameter; (**c**) SAR values at three field frequencies in the case of full and empty dimers; and (**d**) images of full and empty dimers obtained through the etching out of gold nanoparticles [127]. Figure 5 (**IV**) is licensed under CC BY-NC 3.0.

Another solution for increasing the SAR values is the manufacture of magneto-plasmonic NPs. In this case, the magnetic part is used for MHT, and the plasmonic part plays the role of a photothermal material that is excited by laser light. We can meet these special structures with high values of SAR due to the simultaneous application of two stimuli (AMF and laser) under the name of hetero-dimers, nano-stars, and nanoflowers [36]. Guardia et al. [127] synthesized iron oxide on top of gold particles and obtained faceted magneto-plasmonic NPs with an average size of 30 nm and a maximum SAR value of 600 W/g at an *H* × *f* product of 4.4 × 10^9^ A/(ms). The authors concluded that dimers do not lose their heating properties when applying the AMF. Das et al. [82] made Ag@Fe_3_O_4_ nanoflowers with a SAR value of 47 W/g at 9.8 × 10^9^ A/(ms) and 170 W/g when a 442 nm laser (0.52 W/cm^2^) was combined with MHT. Espinosa et al. [128] prepared Fe_3_O_4_ seeds and Au growth with nano-star shapes. MHT efficiency was investigated in an AMF condition of 1.8 × 10^10^ A/(ms), which is not considered suitable for clinical research, and SAR values of 634 W/g were achieved. This effect was combined with that of a 680 nm NIR laser (0.3 W/cm^2^) under safety limits suitable for skin tissue. It can be concluded that these types of composite MNPs can exhibit increased values of SAR, MHT therapy can be combined with photothermal therapy, and enhanced and accelerated effects of cancer cell death are possible. Figure 5 presents the main strategies adopted in improving MHT heat efficiency. 

All the strategies discussed in this section can be adapted for GBM treatment, but it is essential to consider safety and material toxicity for each combination. The establishment of safe combinations that can be applied in clinical trials requires numerous in vivo studies. The development of biodegradable MNPs, which can be eliminated through various routes from the human body, is desirable to mitigate the potential intoxication and side effects associated with different metals. The use of magneto-plasmonic NPs appears to be a promising advancement in cancer therapy, as it allows for the combination of two treatment strategies, potentially leading to more effective outcomes.

## 3. In Vitro and In Vivo Studies for MHT Application in the GBM Treatment

### 3.1. In Vitro Studies

The anticancerogenic effect of the MHT on GBM has been intensively investigated in the past years. One of the most important advantages of this treatment over its counterparts is tumor cell thermosensitivity compared to healthy cells when an AMF is applied. Irreversible oncological cell respiration damages are produced when the temperature is higher than 42 °C, resulting in cell apoptosis since, in the case of healthy cells, temperatures of about 55 °C produce a similar effect. Hanini et al. [129] investigated the thermosensitivity effect on malignant glioblastoma cells (U87-MG) and human endothelial cells (HUVEC) under MHT conditions (AC magnetic field strength of 23.10 kA/m and frequency of 700 kHz). They applied the alternative magnetic field for 1 h in the presence and absence of γ-Fe_2_O_3_ MNPs with a diameter of 10 nm, coated with polyol, and in a superparamagnetic state. Both types of cells, HUVEC and U87-MG, were incubated in combination with 50 μg/mL for 4 h. In order to estimate the MNP quantity internalized by the two cell lines, magnetophoresis and X-ray fluorescence (XRF) spectroscopy were performed. It was noticed that the malignant cells absorbed double MNP quantities compared to healthy HUVEC (magnetophoresis: HUVEC (4.70 pg per cell ± 0.77), U87-MG (8.02 pg per cell ± 0.84); XRF: HUVEC (4.98 pg per cell ± 1.97), U87-MG (8.45 pg per cell ± 3.34)). Hyperthermia measurements evidenced a temperature of 42 °C obtained for both cell lines and SAR values of about 114 W/g ± 21 for HUVEC and 178 W/g ± 37 for U87-MG. Fluorescence microscopy showed that, in the absence of MNPs under AMF action, cell death was less than 10%, while when MNPs were used, this value increased to 20% and 56% in the case of HUVEC and U87-MG cells. The main conclusion of this study was that cancerous cells internalized MNPs much better than healthy endothelial cells and generated a higher SAR value that contributed to cancer cell apoptosis. It can be noticed that this study led to the observation that MHT is a suitable tool for glioma cell annihilation by damaging relatively low amounts of healthy cells. In this way, the tumor can be locally destroyed without important loss of endothelial surrounding tissue due to cell thermosensitivity.

It is well known that to achieve high SAR values, the magnetic properties of the MNPs, such as anisotropy energy, saturation magnetization, and coercivity, must be modified and enhanced through doping operations. In this direction, a study developed by Hanini et al. [130] prepared Zn-substituted ferrite MNPs (Zn_0.9_Fe_2.1_O_4_) to be used for MHT in vitro analysis on U87-MG malignant glioma cells, as described in [129]. The immortalized human glioblastoma U87-MG and healthy umbilical vascular endothelial HUVEC cells were cultured single or in combination with 50 μg/mL MNPs in a DMEM medium. The average size of the MNPs was about 11 nm based on X-ray diffraction and high-resolution electron microscopy. In addition, a classical cubic spinel-type phase was noticed. A saturation magnetization of about 12 emu/g was determined for a temperature of 310 K and a magnetic field strength of 50 kOe. This value was in good accordance with those provided by the literature regarding zinc ferrites. The specific power losses were measured based on magnetocalorimetry, and a value of 36 W/g was obtained. The 20 g/L MNPs were dispersed in distilled water and induced a temperature of about 38.6 °C under the influence of a standard AMF field (289.7 Oe and 700 kHz). When the MNPs were incubated together with glioma cells for 4 h, a much higher temperature increase was determined, and it was concluded that 41.5 °C was sufficient to induce the malignant cell apoptosis. 

Sanz et al. [131] performed an in vitro investigation on human neuroblastoma SH-SY5Y cells by comparing the effect of MHT with that of exogenous heating (EHT) sources. The cells were loaded with different concentrations of PEI-MNPs (10 μg/mL ÷ 100 μg/mL) and then packed into pellets, which simulated an oncological tumor environment. The exogenous heating consisted of a water bath that allowed for reaching a maximum temperature of 56 °C for 30 min. The authors noticed that the MHT produced the same effect of cellular death as hyperthermia generated by exogenous sources and required a much-reduced temperature of 6 °C lower. This fact is of utmost importance if one considers the health of the tissue surrounding a tumor because the increased temperature could determine the unwanted effect of cell death. Supplementarily, it was concluded that the cell death pathways induced by MHT and EHT are quite similar, consisting of destruction and permeabilization of the cell membrane due to an intense heating phenomenon. This study evidenced that MHT is a suitable tool that can have a high potential in the destruction of solid tumors of neuroblastoma cells, and it is very helpful for metastatic patients. Ito et al. [132] demonstrated that heat shock protein (HSP) 70 is induced by MHT and generates antitumor immunity in T-9 murine glioma cells [133]. The authors considered that classical MHT treatments can be safely performed at about 42 °C, but this approach cannot solve the problem of antitumor immunity by itself. If the MNP effect is combined with HSP70 release, fast necrosis of the oncological tissue is evidenced. The two studies mentioned above [132,133] presented the use of magnetic liposomes, which act based on cationic interactions and exhibit increased cellular affinity due to the electrostatic forces that occur between them and the negatively charged phospholipids that exist in the cell membrane.

Another important strategy described in the literature is the application of magnetic gel composite. In most cases, MNPs are introduced into polymer hydrogel matrices. Meenach et al. [134] manufactured poly(ethylene glycol) (PEG)-based magnetic hydrogel and tested its heating efficiency on M059K glioblastoma cells. This study’s main finding was that different heating grades can be achieved by modifying the magnetic field strength of an external AMF. The magnetic gels reached a hyperthermic (42 °C ÷ 45 °C) or a thermoablative (60 °C ÷ 63 °C) temperature. The authors demonstrated the efficiency of the developed magnetic biomaterial in killing the glioblastoma cells only in the thermoablative temperature range but explained that their future studies would include the hyperthermia range combined with drug delivery methods.

Green synthesis methods were involved in the context of innovative manufacturing methods. Ramirez-Nunez et al. [135] developed Fe_3_O_4_@γ-Fe_2_O_3_ nanoparticles coated based on polyphenol using *Cinnamomum verum* and *Vanilla planifolia*. These natural extracts played a double role; firstly, they acted as reducing agents due to their phenolic groups, and, secondly, they bonded with the help of OH groups on the MNPs’ surfaces. Different saturation magnetizations were achieved at about 70.84 emu/g (*Cinnamomum verum*) and 59.45 emu/g (*Vanilla planifolia*). The authors investigated the potential effect of the developed MNPs to be used as magnetic heaters in cancer treatments. In this direction, they performed in vitro studies on immortalized brain microglia murine cell line BV-2 cultured in standard conditions in Dulbecco’s modified Eagle’s medium. The cells were cultured together with 25, 50, and 100 μg/mL. Good cell viability was established in each case. For MHT experiments, only the 100 μg/mL concentration samples were chosen, and, by applying an AMF with a field amplitude of 300 Gs and a frequency of 570 kHz, a temperature of 46 °C was achieved, being followed by the cell death. The authors concluded that the manufactured MNPs were eco-friendly, highly efficient for MHT use, and biologically safe. This research highlights the importance of eco-consciousness in the development of new medical technologies.

A study developed by Hamdous et al. [136] showed the importance of uncoated (M uncoated and MC) and biocompatible coated magnetosome minerals extracted from magnetotactic bacteria. For coating, the authors used polyethyleneimine (M-PEI), chitosan (M-Chi), and neridronate (M-Neri). The MNP heating efficiency for MHT was investigated using mouse glioblastoma cells (GL-261) and rat glioblastoma cells (RG-2). An AMF with a frequency of 198 kHz and strength varying between 34 mT and 47 mT was applied. In the case of PEI- and chitosan-coated samples, the SAR was estimated to have a maximum value of 125 W/gFe. A reduced rate of cellular internalization was established for chitosan-coated samples, while the PEI-coated ones were strongly associated with cells and modulated by the AMF. It was concluded that the highly biocompatible magnetosomes are adequate for MHT treatment, as they are linked to a temperature of about 43 °C and enhance cellular toxicity even when rapid sedimentation of the particles occurs. Gupta and Sharma [137] functionalized Fe_3_O_4_ MNPs with stevioside (STE-Fe_3_O_4_). They considered the natural glycoside to be an excellent surfactant used to control the particle size and, in this way, to modulate their magnetic properties. Cellular uptake efficiency and material biocompatibility were investigated in rat C6 glioma cells. The study was based on a comparative analysis made on MNPs coated with polysorbate-80(P-80-Fe_3_O_4_) and oleic acid (OA-Fe_3_O_4_). After an AMF with a frequency of 405 kHz and an amplitude of 168 Oe was applied, the authors noticed that the stevioside-coated samples exhibited the highest temperature rise, inducing glioma cell death after 30 min by reaching a temperature of 43 °C. The main conclusion of this study was that using a biocompatible coating improved the cell uptake of the MNPs and increased the retention time. It was concluded that, by coating with different polymers, the hydrodynamic diameter of the MNPs was reduced from 447 ± 15.97 nm (bare Fe_3_O_4_) to 191.5 ± 5.72 nm (P-80-Fe_3_O_4_), 270.4 ± 7.25 nm (OA-Fe_3_O_4_), 56.83 ± 10.76 nm (STE-Fe_3_O_4_/0.5 g), and 49.77 ± 6.98 nm (STE-Fe_3_O_4_/1.0 g). The stevioside coating hindered MNP agglomeration due to its negatively charged surface. Mandawala et al. [138] developed stable and biocompatible magnetosomes coated with poly-L-lysine, oleic acid, citric acid, and carboxy-methyl-dextran. Firstly, the authors isolated and purified the magnetosomes from MSR-1 magnetotactic bacteria. Secondly, as mentioned above, they stabilized the magnetosomes obtained with different chemical substances. Cubo-octahedral MNP cores were obtained and surrounded by a highly biocompatible coating. To investigate the heating efficiency and the potential application in MHT for GBM treatment, the magnetosomes were cultured together with GL-261 glioblastoma cells under the effect of an externally applied AMF (34 mT ÷ 47 mT, 198 kHz). SAR values between 89 W/g_Fe_ and 196 W/g_Fe_ were obtained. Different cell death percentages between 10% and 43% were noticed as a function of the chosen biocompatible coating and the AMF parameters. The authors concluded that the coated magnetosomes could be successfully applied for GBM tumor treatment, and the SAR values and in vivo distribution can be easily optimized to obtain the best-expected results. Rego et al. [139] prepared amino silane-coated iron oxide MNPs for MHT application in the GBM model. They performed an analysis regarding the SAR values and chose various types of AMF with a constant magnetic flux density of 100 Gs, 200 Gs, and 300 Gs and different frequencies between 309 kHz and 557 kHz. The SAR values varied between 3.789 ± 0.137 W/g (309 kHz, 100 Gs) ÷ 11.078 ± 0.403 W/g (557 kHz, 100 Gs), 54.757 ± 1.460 W/g (309 kHz, 200 Gs) ÷ 82.772 ± 3.548 W/g (557 kHz, 200 Gs), and 169.297 ± 5.097 W/g (309 kHz, 300 Gs) ÷ 320.070 ± 22.818 W/g (557 kHz, 300 Gs). For the in vitro investigations performed on the C6 cell line, the authors selected the 300 Gs and 309 kHz or 557 kHz conditions. Based on bioluminescence (BLI) measurements in the cases in which cells were cultured with MNPs and the AMF was applied, a reduction in BLI intensities was noticed (e.g., from (4.365 ± 0.276) × 10^9^ to (1.748 ± 0.112) × 10^9^ photons/s–300 Gs, 309 kHz and from (4.367 ± 0.276) × 10^9^ to (8.730 ± 0.873) × 10^8^ photons/s–300 Gs, 557 kHz). It was concluded that cell death occurred at a high rate under the MNPs and AMF effect and that the developed MNPs have high potential for MHT in GBM treatment.

**Figure 6:**
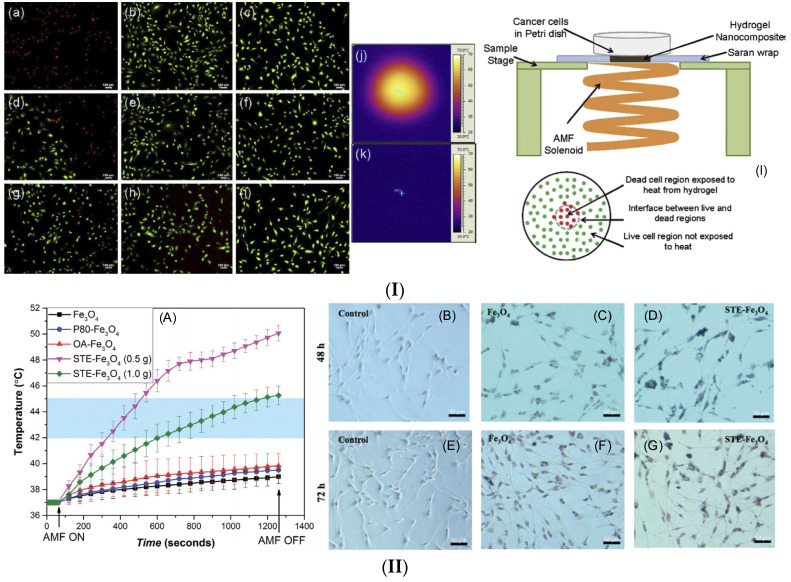

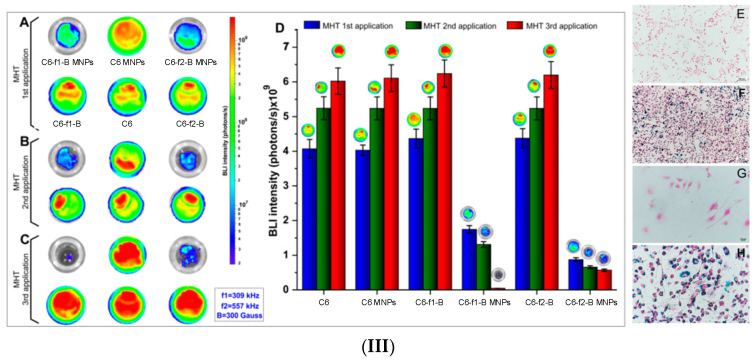
In vitro results of various MHT protocols. (**I**) Heating efficiency test on M059K cells of PEG-based magnetic hydrogel under AMF influence. Fluorescent microscopy images after Life/Dead assay: (**a**–**c**) zone exposed directly to AMF in the center of the Petri dish; (**d**–**f**) zone placed at the boundary between dead and live cells; (**g**–**i**) zone placed near the edge of the Petri dish (First column: sample exposed to PEG-based magnetic hydrogel and AMF; second column: sample exposed to AMF; third column: control sample); infrared (IR) image of cells and hydrogel: (**j**) heated for 5 min; (**k**) under AMF effect for 5 min; and (**l**) schematic representation of the in vitro test. Reprinted from [134]. Copyright (2024) with permission from Elsevier. (**II**) In vitro MHT test on C6 cells based on different types of MNPs. (**A**) Temperature response at an AMF of 168 Gs and 405 kHz for 20 min.; (**B**–**G**) Prussian blue staining of C6 cells for control and incubation with different types of MNPs after 48 h and 72 h (scale bar 50 μm) [137]. Figure 6 (**II**) is licensed under CC BY 4.0. (**III**) In vitro MHT test on C6 cells based on aminosilane-coated Fe_3_O_4_ commercial MNPs. (**A**–**C**) BLI signal analyses after 1, 2, and 3 MHT cycles; (**D**) Histogram of BLI intensities; and Prussian blue and nuclear fast red images: Control: (**E**) 4× magnification; (**G**) 20× magnification; After labeling with 200 μgFe/mL MNPs, (**F**) 4× magnification and (**H**) 20× magnification [139]. Figure 6 (**III**) is licensed under CC BY 4.0.

In vitro studies are necessary for the first assessment of MNP biocompatibility and potential for MHT application in GBM treatment. All the investigations presented in this subsection were performed on human or animal glioblastoma cell lines and proved the efficiency of MHT in inducing cell death. Figure 6 presents some in vitro results after the application of different MHT protocols.

Table 3 emphasizes the main studies regarding MHT efficiency in glioblastoma treatment.

### 3.2. In Vivo Studies

In vivo studies are necessary to investigate the treatment effect directly on an animal model and to estimate the outcome of magnetic hyperthermia. Many literature studies have considered GBM treatment based on different MNPs and, most of all, have used murine animals. One of the first in vivo investigations seen in the literature was developed by Yanase et al. [141], who investigated intracellular hyperthermia based on Fe_3_O_4_ liposomes applied to reduce a solid glioma tumor artificially induced in the left femoral region in the case of rats. An AMF with therapeutic characteristics was applied, and the rats were divided into three groups with different numbers of AMF cycles as follows: control group I—no AMF cycle, group II—one irradiation cycle applied for 30 min, group III—two irradiation cycles, and group IV—three irradiation cycles. Complete tumor regression was obtained in the case of group IV in a higher percentage compared with the other groups (e.g., 87.5%—group IV, 60%—group III, 20%—group II). In addition, for groups III and IV, widespread distribution of magnetic liposomes was noticed in combination with necrotic cells in the tumor area. It was concluded that the most efficient way in which the treatment could be applied consisted of three cycles of AMF irradiation. The same research group developed, in [142], an interesting analysis regarding antitumor immunity induction after MHT application. They used the heating property of Fe_3_O_4_ positively charged liposomes in T-9 rat glioma tumors created through subcutaneous injection on both rat femurs. The left femur was chosen as the treatment site to administer the magnetic liposome solution. In this study, the animals were assessed into two groups: the first one with no AMF application and the second one characterized by 30 min irradiation in three consecutively applied cycles at 24 h intervals. It was noticed that the glioma tumor localized on the left side in the cases in which the AMF was applied completely disappeared. In addition, annihilation of the glioma cells of the right-side tumors was also evidenced. After that, the healed animals were injected again with T-9 cells 3 months after MHT sessions. It was observed that after transient tumor development, all the cancerous cells disappeared. The authors underlined the importance of their findings by concluding that, in some cases, antitumor immunity can be induced by magnetite liposome injection. This observation is of utmost importance because it proved the capacity of living organisms to heal themselves based on acquired immunity after a full cycle of MHT was applied. Jordan et al. [143] manufactured two magnetic fluids based on aminosilane- and dextran-coated iron oxide MNPs. They implanted RG-2 cells into 120 rats’ brains to induce glioblastoma multiforme tumor apparition. The animals were allocated to 10 groups containing 12 rats. Control was included. The protocol treatment consisted of one intratumoral injection. On days 4 and 6 after tumor induction, an AMF with a variable magnetic field strength up to 18 kA/m and a frequency of 100 kHz was applied. It was noticed that the animal group treated with aminosilane-coated iron oxide magnetic fluid exhibited a higher survival rate of 4.5-fold compared to control and dextran-coated MNP groups. Immunohistochemical analyses demonstrated the existence of large necrotic zones placed in the vicinity of amino silane-coated MNPs. The authors concluded that the coating of the MNPs had a decisive role in tumor reduction and prolonged rate survival. It can be foreseen that the development of biocompatible coatings can be an important research direction.

Besides finding proper coatings for MNPs to increase their heating efficiency and prohibit their agglomeration, another strategy described in the literature consists of combining heat-induced therapeutic gene expression or specific antibodies with magnetic hyperthermia, which seems to provide excellent results in inducing cancer cell death. Ito et al. [132] showed that the expression of heat shock protein 70 (HSP 70) generated antitumor immunity in the T-9 cell glioma tumors. As presented in Section 3.1., the authors purified the HSP70-peptide from the tumor after a hyperthermia treatment was applied. They noticed that, in the case of F344 rat animal models, which were previously immunized with T-9-derived HSP70, tumor growth was almost totally reduced. T-9 cells combined with magnetite cationic liposomes were implanted in the animal body, and tumor rejection assays were used to demonstrate that antitumor immunity was developed. It was concluded that this combined therapy dedicated to the treatment of a stereotactically implanted tumor in the subcutaneous zone led to important tumor immunity, being very helpful in GBM treatment. Le et al. [144] prepared tumor-specific magnetoliposomes that were conjugated with an antibody fragment. They performed an in vivo experiment based on glioma-harboring mice, which were injected with modified magnetoliposomes and then irradiated under an alternating magnetic field. A magnetic hyperthermia effect occurred, and a temperature of 43 °C was achieved in the tumor zone. The authors noticed after 2 weeks a regression of the tumor. The importance of this study consists of the fact that the problem of low amounts of magnetic heater accumulation in the tumor zone was solved through unique antibody-conjugated magnetoliposomes, which proved to have high heating potential under an AMF influence. Ito et al. [145] applied heat-induced therapeutic gene expression once again, but this time, they combined *TNF–*α gene therapy with MHT based on the stress-inducible promoter *gadd* 153 and magnetite cationic liposomes. An AMF with a frequency of 118 kHz and a magnetic field strength of about 384 Oe was applied during one magnetic irradiation session of 30 min. In vivo studies were conducted on mice with artificially induced U251-SP human glioma tumors. The authors noticed a powerful cell-killing effect on the cancerous cells. Although *TNF–*α was not able to cause complete cell death, a strong tumor-growth-diminishing effect was evidenced in combination with the heat generated by the MNPs. The magnetoliposomes used alone induced cell death, but tumor growth was not entirely arrested. It was concluded that this combined strategy should be considered due to the *TNF–*α property to inhibit neovascular apparition and to damage GBM blood vessels. In this way, the nutrients and oxygen that fed the tumor were not available anymore, and based on the heating effect of MNPs, cell death occurred with no new cancer cell apparition. Unfortunately, it is worth mentioning that *TNF–*α gene therapy has important side effects due to systemic toxicity related mainly to hepatocellular degeneration. The authors concluded that their treatment route can be considered a powerful tool against GBM, but the protocol must be carefully applied due to the important drawbacks of *TNF–*α therapy.

An innovative laboratory study developed by Ohno et al. [146] evidenced hyperthermia’s positive effect as a single treatment option in malignant glioma based on stick-type carboxymethylcellulose (CMC)-magnetite. The authors implanted CMC-magnetite into a T-9 rat glioma model. The animals were irradiated in an AMF (380 Oe and 88.9 kHz) for 30 min daily. Three groups of rats were analyzed. Group I was treated with three irradiation sessions, group II had only one session, and the control group had no AMF and only the CMC-magnetite stick was applied. The developed stick implant was characterized by its facile use, the possibility of treating a specific zone of the brain, and exhibiting an optimal concentration of Fe_3_O_4_. The highest survival rate and the most efficient way to attack the glioma cells was the AMF application by three times due to its strong hyperthermic effect, as well as strong diffusion of the MNPs through the glioma tumor. It can be concluded, as seen in many studies in the literature, that the application of multiple irradiation sessions is a promising way to conduct MHT treatments dedicated to GBM.

Magnetosomes isolated from magnetotactic bacteria represent an important research direction identified in the literature. This approach was used for MHT application in the case of GBM treatment. We will present some of the most important studies based on this concept. Fevre et al. [31] used magnetosomes to reduce and alleviate GL-261 mouse glioma cell-induced tumors in C57/BL6 mice. They divided the animals into four groups as follows: group 1 (received magnetosome injection, 11 to 15 magnetic irradiation sessions (MSs) in an AMF with a frequency of 198 kHz and strength between 11 and 27 mT), group 2 (received a magnetosome injection without AMF irradiation), group 3 (received classical Fe-based MNPs and 7 to 15 MSs in an AMF with a frequency of 198 kHz and strength between 22 and 31 mT during the first MS and between 22 and 27 mT during the second MS), group 4 (received Fe-based MNPs without AMF irradiation), group 5 (received glucose injection and 4 to 10 MSs in the AMF with the same frequency and average strength of 27 mT), and group 6 (received only 50 μL of 5% glucose suspension). Groups 2, 4, 5, and 6 were considered as control groups. The best survival rate and higher tumor reduction was achieved for group 1. In the case of magnetosome administration, the survival rate of the animals was estimated at 50%. They were cancer-free in 5 weeks in the case of group 1 for the magnetic field product *H* × *f* = 2 ÷ 4 × 10^9^ A/(ms). Above this limit, the MHT treatment was considered dangerous due to eddy current apparition [33]. By comparing the efficiency of magnetosomes with that of Fe-based MNPs, it was concluded that the magnetosomes exhibited higher SAR values (e.g., SAR_magnetosome_= 40 W/gFe, SAR_Fe-based MNPs_ = 26 W/gFe), an increased retention time inside the tumor environment (e.g., 5 days—magnetosomes, 1 day—Fe-based MNPs), and increased temperature (e.g., 46 °C—magnetosomes, 43 °C—Fe-based MNPs). The authors observed that reducing the tumor dimensions without magnetosomes was also possible when AMF sessions were applied. A possible explanation consists of the fact that tumor ischemia or antitumor immune response occurred. The main conclusion of this study was that the developed magnetosomes are an ideal candidate for MHT application to cure glioblastoma tumors.

Alphandery et al. [147] synthesized MNPs from magnetotactic bacteria and proved their efficiency regarding complete reduction in intracranial U87-Luc glioma in mice. The animals were injected with about 13 μg of magnetosome chain per tumor mm^3^ and introduced in an AMF with 198 kHz and 30 mT in therapeutic conditions [146,148]. The number of magnetic irradiation sessions was between 12 and 15 for 30 min each. Full tumor destruction was achieved for 40% of mice with no tumor metastasis apparition. The authors noticed that the developed magnetosomes were able to release a high level of endotoxins (1400 ÷ 8400 EU/mL/mg) compared with classical iron oxide MNPs (<50 EU/mL/mg) under the effect of the applied AMF, a fact that was considered beneficial for cancerous cell death through the apoptosis phenomenon. An interesting result was found in the case of magnetosomes, which infiltrated only a percentage of 10% inside the tumor, proving that an immune response occurred. It was concluded that biocompatible magnetosomes are very efficient in obtaining glioma remission under AMF conditions, and the main physical mechanism responsible for oncological cell death was the large quantity of heat that was generated based on the MHT phenomenon, as well as due to endotoxin release. One can observe that this type of treatment is suitable for infiltrating tumors such as GBM because, in these cases, full tumor coverage with MNPs is almost impossible to achieve during in vivo treatments. The study mentioned above was continued in [149]. Alphandery et al. coated the mineral magnetosomes with poly-l-lysine to increase the biocompatibility of the MNPs. Superior results compared to the authors’ previous investigations were achieved due to the improved ferrimagnetic behavior of the magnetosomes and higher heating capabilities. In this study, the animals underwent 27 magnetic sessions (30 min for each MS) under the effect of an AMF (frequency of 202 kHz; magnetic field of 27 mT). It was observed that, using the coated magnetosomes, a temperature of 42 °C was obtained in the cancerous U87-Luc tumors with a volume of 1.5 mm^3^. The outcome of the above-described treatment protocol consisted of healing in a percentage of 100% of the inoculated mice after 68 days. Bioluminescence intensity measurements of living GBM cells showed important tumor volume reduction starting on day 7 after surgery and ending with day 35. It was also extraordinary that the animals were still healthy and alive on day 350 after tumor apparition. The authors concluded that the mice were fully cancer-free, and no metastases were reported. It can be foreseen that the developed coated magnetosomes are an important and promising candidate for GBM treatment and could be successfully introduced as an alternative in clinical trials.

**Figure 7:**
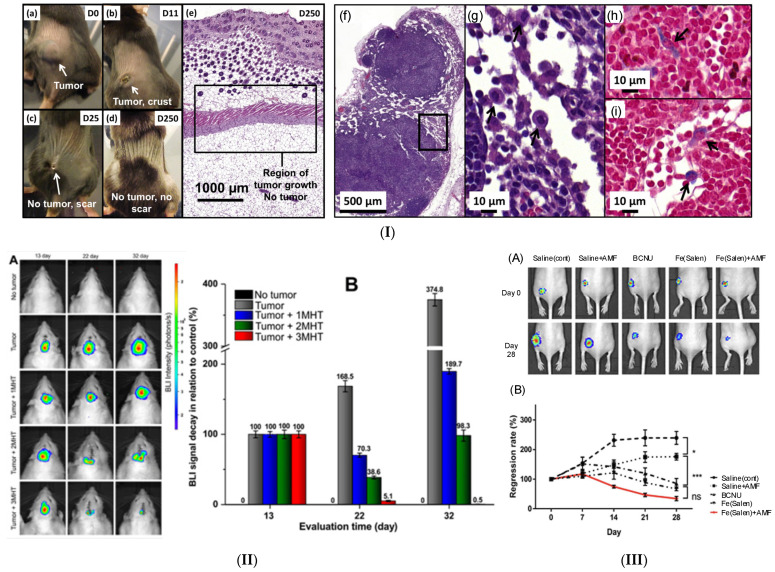
In vivo results of MHT treatments. (**I**) In vivo result of biocompatible magnetosomes used in MHT protocol for GBM: (**a**–**d**) photographs of the tumor site on day 0 (D0), 11 (D11), 25 (D25), and 250 (D250) after MHT session; (**e**) H&E section of skin in day 250 from the initial place of the tumor with no cancer cells; sample of a lymph node collected from a cured mouse after 15 MHT sessions in day 250; (**f**) H&E lymph node; (**g**) H&E zoom, which evidences the macrophage presence (black arrow); and (**h**,**i**) Perls’ Prussian blue zoom sections presenting a blue color in the macrophage cytoplasm (black arrow), proving full elimination of the magnetosomes [31]. Figure 7 (**I**) is licensed under CC BY-NC 4.0. (**II**) In vivo result of aminosilane-coated MNPs used in multiple MHT irradiations: (**A**) BLI images of the tumor at day 13, 22, and 32 and different numbers of MHT applications; (**B**) BLI signal variation in relation to control versus evaluation time [139]. Figure 7 (**II**) is licensed under CC BY 4.0. (**III**) In vivo investigation of MHT and chemotherapy based on Fe(Salen) MNPs: (**A**) images of mouse leg tumor at day 0 and day 28 in the case of different treatment protocols; (**B**) regression rate of tumor volume measured at different days of treatment (n = 6; ns, not significant; * *p* < 0.05; *** *p* < 0.001) [150]. Figure 7 (**III**) is licensed under CC BY 4.0.

All the in vivo studies presented in this subsection were performed on murine animals and involved human or animal glioma-cell-line-induced tumors. It can be noticed that different treatment protocols were proposed (Table 4), and some of them were successful in reducing tumor volume or in establishing so-called immunity against glioma cells. Their combination with different targeting agents or the manufacturing of magnetosomes proved their efficiency in killing glioma cells, but much more research is necessary to introduce these solutions in clinical trials. Considering all the in vitro and in vivo research, in our opinion, MHT treatment can represent a viable solution to help the patient with GBM. However, some advances in the physical system can be necessary to establish a correct treatment route and to hinder the apparition of eddy currents generating a local overheating phenomenon. All the proposed solutions must be extremely carefully applied with respect to human subject anatomy because of notable differences from murine animals and have to be adapted in order to not exhibit important drawbacks. Figure 7 shows some in vivo results after the application of different MHT treatments.

Table 4 presents a comparative analysis of different magnetic hyperthermia treatments used in glioma murine animal models.

**Table 4:** In vivo studies for MHT dedicated to GBM analysis.

Mnps/Injection Zone	Cell Lines/Number of Inoculated Cells	AMF Conditions–H (Oe), f (kHz), Treatment Time	Number of AMF Cycles and Time between AMF Cycles	Animal Model	Animal Particularities	Ref.
Fe_3_O_4_ cationic liposomes combined with HSP70 gene therapy/Subcutaneous zone/Combined therapy MHT + Immunotherapy	T-9/1 × 10^6^ cells	384 Oe/118 kHz/30 min	1,2,3 cycles/24 h	F344 (Fisher) rats	Female/6-week-old	[132]
Fe_3_O_4_ cationic liposomes/Subcutaneous site placed at left femoral region/Single MHT therapy	T-9/1 × 10^7^ cells	384 Oe/118 kHz/30 min	1,2,3 cycles/24 h	F344 rats	Female/7-week-old	[141]
Fe_3_O_4_ cationic liposomes/Subcutaneous site placed at left and right femoral region/Single MHT therapy	T-9/1 × 10^7^ cells	384 Oe/118 kHz/30 min	3 cycles/24 h	F344 rats	Female/7-week-old	[142]
Specific tumor antibody fragment conjugated with Fe_3_O_4_ magnetic liposomes/Subcutaneous femoral zone/Combined therapy MHT + Immunotherapy	U251-SP/2 × 10^5^ cells	384 Oe/118 kHz/30 min	3 cycles/24 h	KSN nude mice	Albino athymic nude mice/Female/4-week-old	[144]
Aminosilane-coated MNPs, dextran-coated MNPs/Subcutaneous anterior part of the brain/Single MHT therapy	RG-2/1 × 10^5^ cells	0 ÷ 226.2 Oe/100 kHz/40 min	1 cycle	F344 rats	Male	[143]
MNPs functionalized with aCD133/Subcutaneous in mouse striatum/Combined therapy MHT + Immunotherapy	CT-2A/2 × 10^5^ cells	2513.27 Oe/very low frequency field/2 h/day	7 cycles in 7 days	C57 mice	C57 black 8 mice/Female/22-week-old	[151]
Carboxymethylcellulose (CMC)–Fe_3_O_4_ MNPs/Single MHT Therapy	T-9/5 × 10^6^ cells	380 Oe/88.9 kHz/30 min	3 cycles/24 h	F344 rats	Female/4-week-old	[146]
Magnetoliposomes/Subcutaneous in the animal back/Single MHT Therapy	GL-261/1 × 10^7^ cells	Group 1: 11 ÷ 27 mT/198 kHz/-; Group 3: 22 ÷ 31 mT, 22 ÷ 27 mT/198 kHz/-; Group 5: 27 mT/198 kHz/-	Group 1: 11 and 15 sessions; Group 3: 7 and 15 sessions. Group 5: 4 to 10 sessions	C57/BL6 mice	Female/6-week-old	[31]
Chains of magnetosomes/Intracranial inoculation of glioma cells based on craniotomy/Single MHT Therapy	U87-Luc/2 × 10^5^ cells	30 mT/198 kHz/30 min	12 ÷ 15 cycles	CD-1 nude mice	Female/7-week-old	[147]
Non-pyrogenic magnetosomes coated with poly-l-lysine/Intracranial inoculation of glioma cells based on craniotomy/Single MHT Therapy	U87-Luc/2 × 10^5^ cells	27 mT/202 kHz/30 min	27 cycles	Charles River pathogen-free athymic nude mice	Female, 18 g/5-week-old	[149]
Fe_3_O_4_ magnetic liposomes combined with *TNF*–α gene therapy/Right flank/Combined therapy MHT + Immunotherapy	U251-SP/3 × 10^7^ cells	384 Oe/118 kHz/30 min	1 cycle	Athymic nude mice	Female/4-week-old	[145]
Fe_3_O_4_/Subcutaneous space/Single MHT therapy	U251/1 × 10^7^ cells	-/200 kHz/60 min	2 cycles	Nude mice	Male	[152]
γ–Fe_2_O_3_ coated with dextran/Bregma region/Single MHT therapy	C6/8 × 10^6^ cells	138.2 Oe/150 kHz/20 min	1 cycle	Wistar rats	Male	[153]
Amino-silane coated superparamagnetic Fe_3_O_4_ nanoparticles/Motor cortex/Single MHT therapy	C6/1 × 10^6^ cells	300 Gauss/309 kHz/30 min	3 cycles	Wistar rats	Male	[139]
Fe(Salen)/Leg/Combined therapy MHT + Chemotherapy	U251/1 × 10^7^ cells	31 mT/280 kHz/-	1 cycle	Balb.c nu/nu mice	Female/6-week-old	[150]

## 4. MHT Therapy in Combination with Other Therapies

MHT was used in clinical trials performed on patients with GBM in combination with other therapies. Some of the most important associations are MHT and radiotherapy (RT), MHT and chemotherapy, MHT and light-based therapy, and MHT and immunotherapy. In the previous section, we have already named a few studies and proved that a combined treatment route can be much more efficient than MHT used as a single therapy. In this section, we will provide the action mechanisms that occur and kill cancer cells and some examples of in vitro or in vivo studies extracted from the literature. 

MHT was combined with radiotherapy due to the presence of abnormal blood vessels at the tumor site compared to healthy tissue. In these cases, a hypoxic tumor environment characterized by a lack of oxygen has an important contribution to transforming the oncological cells into radioresistant ones [154,155]. Combining the RT with MHT can overcome this radioresistance phenomenon [156,157] because heat apparition usually strongly influences radioresistant cells [158,159]. The abnormal vasculature inside the tumor does not have the normal blood vessel ability to expand and increase the speed of blood to regulate heat, so the generated heat through MHT remains inside the tumor [160,161]. In addition, it is well known that lactic acid accumulation occurs under hypoxic conditions, and the pH decreases, making the cancer cell more prone to the presence of heat [162]. In this way, the MNP’s heat efficiency is enhanced by coupling with RT, and the treatment becomes stronger and much more efficient [36]. This approach was one of the most investigated in the literature and has been used in pre-clinical and clinical studies [163,164]. Studies have revealed that the time interval between the application of irradiation and MHT remains one of the key parameters in obtaining a successful result. Decreasing the time between each application can maximize cancer cell death [165] since a difference higher than 4 h between RT and MHT increases the radiosensitization of the cell under the heat effect [166]. Supplementarily, a so-called thermo-tolerance phenomenon due to mild MHT conditions was evidenced in [167], which induced temporary resistance to heat treatment. Usually, during this combined therapy, the tumor region is firstly irradiated; after that, MNPs are injected, and, as a third step, an AMF with therapeutic characteristics is applied. In clinical trials performed on GBM patients, 15 nm aminosilane-coated spherical iron oxide MNPs were successfully applied [168,169]. In Table 5 some of the most relevant studies found in the literature related to RT + MHT combined therapy are provided [169,170,171].

Another highly investigated treatment strategy consists of a combination of chemotherapy and MHT. It is generally accepted that chemotherapy can be characterized by a lack of specificity concerning tumor particularities and can determine important side effects [7,168]. In most cases, reduced drug doses are used and can lead to insufficient cancer cell death. Many literature studies [7,172,173] evidenced the efficiency increase in chemotherapeutic medicines due to MHT, and this is usually called thermo-chemo-sensitization. Under the influence of heat, the perfusion and permeability of the tumor blood vessels are enhanced, and drugs at the tumor site spread much more quickly and efficiently [36]. In addition, high cancer cell membrane permeability is accompanied by a reduction in DNA classical repairing mechanisms in the MHT temperature range between 42 °C and 47 °C, so, in this way, cell uptake and drug action mechanisms are improved [168,174,175]. Also, by using chemotherapeutic drugs, which act locally, the number of MNPs can be adjusted in order for the therapeutic temperature to have a maximum value of 43 °C with no significant harmful effects on the healthy surrounding tissue and reduced toxicity of the MNPs. It can be observed that intense research is being conducted to manufacture MNP-based platforms that have magnetic material heating properties and can release and control chemotherapeutic drugs efficiently. Solutions such as thermo- and/or pH-responsive materials have been proposed. In this direction, the heat produced by the MHT phenomenon can be used as an external stimulus that can start the drug-release process. Supplementarily, the tumor has a particular microenvironment, which favors controlled release through intratumoral stimuli such as hypoxic and pro-oxidant states or acidic pH [176,177,178]. Unfortunately, only a few studies have associated MHT with intratumoral-stimuli release, while heat-mediated drug delivery is much more investigated.

Regarding the latter, MNPs are functionalized with coatings that permit a thermal-dependent interaction between drugs and coating and can be controlled through heat induced by MHT. In these cases, the drug is released based on diffusion. Another class of materials involves inserting thermal-sensitive material into the coating/shell of MNPs to block or encapsulate drug molecules. 

Considering the systems based on direct thermal-sensitive interaction between MNPs and chemotherapeutic agents, combinations between MNPs and thermo-responsive (TR) polymers were developed. In polymers with a lower critical solution temperature (LCST), hydrogen bonds with water or hydrophilic drug molecules are formed. With the temperature increase above LCST, the hydrogen bond geometry is modified, generating a mechanical contraction of the polymer, which can release the drug [36]. Mai et al. [179] performed surface functionalization between Fe_3_O_4_ cubic MNPs and oligo ethylene glycol methyl ether methacrylate (POEGMA) at a magnetic field strength intensity of 11 kA/m and a frequency of 105 kHz. The authors loaded the TR polymers with doxorubicin, and the drug was released in in vivo conditions in the case of a mouse animal model. Another candidate was considered—polynucleotide, which exhibits hydrogen donors and acceptors. In [180], Li et al. functionalized Fe_3_O_4_ with a polynucleotide (5′-AAAAAAAAAAAAAAA-3′, A15) and bis amino polyethylene glycol (PEG). The amine group hindered functionalization between the developed magnetic system and antibody anti-HER2, permitting the delivery of the 5-fluorouracil (5-FU) drug to a cancerous tumor in mice. Another approach in the literature consists of host–guest chemistry, defining the host as MNP surface functionalization with cyclic macromolecules and drugs as the guest linked through interaction forces, not chemical bonding. Unfortunately, none of these solutions were applied to GBM treatment, but they should be exploited as viable possibilities in the future. An important strategy described in the literature is the production of a system based on a gating/trapping process, which represents a combination of mesoporous silica, a self-assembled polymer/phospholipid platform, or injectable hydrogels and MNPs, which play the role of a hosting template used to release a desired drug. The system based on mesoporous silica is characterized by dose control in temporal and spatial coordinates, being a proper candidate for use in conjunction with MHT, but much more research must be performed due to a lack of in vivo investigations [36]. Nanoformulation based on liposomes is the only platform approved for clinical use [181,182,183]. Liposomes exhibit a vesicular structure and can be loaded with hydrophilic or hydrophobic molecules. In addition, their membrane has a thermo-sensitive lipidic composition that can be disrupted around 42 °C [184]. Thermosensitive magneto-liposomes are obtained by introducing MNPs in lipidic structures [185]. Babincova et al. [186] made magneto-liposomes composed of DPPC/CH/DSPE-PEG200, in which encapsulated MNPs were loaded with doxorubicin. An in vivo study was performed on a mouse glioma model irradiated in an AMF (field intensity of 30 kA/m, frequency of 3.5 MHz) twice weekly for 28 days. Complete tumor regression was achieved. Polymersomes are characterized by a low micelle concentration, being much more thermodynamically stable than liposomes [187]. They can be easily combined with MNPs. Numerous studies related to these systems in combination with MHT have proven the treatment’s efficiency on different cancer cell lines [188,189]. As in the other cases, the necessity of investigation performed on GBM cell lines or animal models needs to be more analyzed, giving more space in science for development in this direction. Injectable hydrogels represent a viable way to deliver MNPs and chemotherapy. Usually, the gel is first made and then subcutaneously implanted at the tumor site. This gel blocks the MNPs and drugs inside it, preventing their leaching near the tumor. In practice, different types of hydrogels are used, such as polymeric-, α-cyclodextrin-, peptide-, and lipogel-based. One of the most investigated materials combined with MHT are polymers and α-cyclodextrin that are used for in vivo models [190,191].

It can be noticed that the heat effect on different chemotherapeutic drugs was reported to be synergistic (taxanes, fluorouracil), supra-additive (platinum-based agents and alkylating drugs), and threshold behavior (doxorubicin) [192]. Table 5 presents some examples found in the literature related to the combination of MHT and chemotherapy [150,193,194].

MHT combined with light-based therapy includes the association with photothermal therapy (PTT) and photodynamic therapy (PDT). Sometimes, MHT based on MNPs can exhibit an important disadvantage, which is low power absorption efficiency in the case of tumors localized deep inside the tissue [36]. To increase the quantity of heat, applying a strong magnetic field that is not included in the therapeutic range or increasing the MNP quantity with related toxicity issues is required [195,196]. The PTT exploits the light possibility to induce heat. This phenomenon is based on the properties of some materials that absorb light; then, electrons are excited to a superior energy state, and non-radiative processes such as heat release appear. In practice, the light-absorbing materials are phthalocyanine, cyanine, rhodamine groups (PDT) [197], semiconducting materials (graphene or carbon nanotubes), and nanoparticles that can absorb the light through a Surface Plasmon Resonance phenomenon, which is associated with coherent oscillations of metallic electrons localized in the conduction band that are in resonance at light frequencies (PTT) [198,199]. One can enumerate metals such as gold (Au), silver (Ag), and palladium (Pd) or chalcogenides (Cu_2-x_E, with E sulfur (S), selenium (Se), or tellurium (Te)). The last-mentioned materials can absorb near-infrared light (NIR) in the wavelength domain of 650 nm ÷ 950 nm and convert it into heat [200,201,202]. Unfortunately, PTT as a single therapy cannot be applied to tumors localized deep within healthy tissue due to the limited penetration possibility of NIR light (10 mm) [203]. Combining plasmonic materials with magnetic ones seems to be an appropriate strategy, making it possible for the toxicity associated with MNPs or plasmonic compounds to be reduced to a minimum. In addition, a higher heat value can be achieved and locally applied to accelerate tumor volume reduction. Usually, MNPs are inoculated through intratumoral deposition, and then laser light and AMF irradiation are applied. The combined therapy of MHT and PTT was included in a few studies regarding GBM treatment. Additionally, this dual heating strategy was successfully applied to human squamous carcinoma (A431), prostate cancer (PC3), and ovarian carcinoma (SKOV3) cell lines [204]. The positive results obtained in other types of cancer proved the potential of this combined therapy as an appropriate candidate for GBM’s possible cure. Table 5 provides examples of in vitro investigations related to GBM cell lines and the MHT+PTT combined strategy [140,205,206].

Photodynamic therapy (PDT) has, as its primary mechanism, the apparition at the tumor site of toxic free radicals such as OH^−^, HO_2_^−^, O_2_^−^ and singlet oxygen ^1^O_2_ as a result of photobiological and photochemical processes, which are due to the interaction of a photosensitizer (PS) (porphyrin) and the oxygen existing in living tissue under the effect of a given light wavelength [36,207,208,209]. In clinical trials with FDA approval, a porphyrin-based photosensitizer called Photofrin^®^ is used [210,211]. If PDT is combined with MHT, many reactive oxidative species (ROS) are produced due to PS action and MNPs’ intrinsic toxicity. A main drawback of this combined strategy is that porphyrin remains in the human body for a long time and generates increased light sensibility, so it is recommended that the quantity of this drug be reduced. This is possible when the PDT effect is increased by the heat generated during MHT treatment. This process is obtained by combining the MNPs with a photosensitizer and injecting the resulting material at the tumor site. Then, the tumor will be simultaneously irradiated by light and AMF to activate ROS generation. Table 5 details some studies that use a combination of PDT and MHT to prove its efficiency with glioma cell lines [212,213]. There is a lack of literature regarding PDT and MHT combined therapy in conjunction with in vitro or in vivo studies related to GBM. However, this approach proved to be very efficient in reducing tumor sizes made of SKOV3 xenografts [214] by using a laser (wavelength of 650 nm, laser power of 100 mW/cm^2^) and an AMF (*f* = 111 Hz, *H* = 23.8 kA/m) for 30 min. In addition, Curcio et al. [215] prepared γ-Fe_2_O_3_ nanoflowers with a spiky copper sulfide shell (Fe_2_O_3_@CuS) characterized by an increased near-infrared (NIR) absorption coefficient adequate for PDT and PTT application. The heating efficiency of MHT and PTT was combined with PDT’s unique characteristics to increase the released ROS species. The treatment efficiency was tested based on in vitro and in vivo investigations performed on human prostate adenocarcinoma PC3 cells and on 30 pathogen-free 9-week-old immunodeficient athymic nude mice with surgically induced tumors. During MHT analysis, an AMF with a frequency of 471 kHz and a field of 18 mT was applied. Higher SAR values of about 350 W/g were achieved. For the PTT step, a laser (1064 nm, 0.3 W/cm^2^) was used. The heat produced via PTT was combined with that induced through MHT. For PDT therapy, the laser power was decreased to 1 W/cm^2^ to avoid the phenomenon of ROS signal saturation. The authors observed that, for the in vivo study, the PTT was much more efficient than MHT in the cases in which the tumor was placed in the skin vicinity, while for the tumors localized very deep inside the healthy tissue, MHT was preferred. In addition, applying the PDT strategy led to the highest tumor reduction. Considering the studies performed on cancer cell lines other than glioma, one may assume these combined strategies are a viable solution to address the GBM problem in a much more detailed analysis. Multi-therapeutic strategies are of utmost importance because they benefit from multiple physiological ways to kill cancer cells faster and in a higher amount. 

Last but not least, MHT was combined with immunotherapy in some in vitro or in vivo studies that analyzed the treatment efficiency in a direct relationship with GBM cell lines or solid tumors (Table 5). Cancer immunotherapy enhances the human or animal body to fight against the tumor and even to destroy it [36,216,217,218,219]. In some cases, the immune system cell infiltration into the tumor activates the recruitment of innate and/or adaptive immune system cells, which suppress the tumor action [220]. From the innate immune system cells, one can mention macrophages, dendritic cells, neutrophils, natural killer cells, basophils, mast cells, and eosinophils, and it can be noticed that the action of these cells does not require specific stimulation through antigens [221]. On the other hand, the cells included in the adaptive immune system, such as T and B lymphocytes, need an antigen-presenting cell at the tumor place to be activated and to transform into antigen-specific B or T-cells, which can effectively kill the cancer cells, and to maintain long-time host immunity [222]. In [223,224,225], it is stated that the presence of heat can control and activate the intratumoral immune system cell action, increase interleukin generation, and favor the circulation of immune cells via tumors based on lymph nodes. This fact is due to the dilation of blood vessels under the heat effect in the tumor vicinity. In the cases in which hyperthermia was applied in the neighborhood of cancer tumors, some damage-associated molecular patterns antigens that can be recognized by tumor-associated dendritic cells and macrophages are released [226,227]. One of the most important damage-associated molecular patterns antigens are heat shock proteins, which result from necrotic cells and transform tumor-associated dendritic cells into antigen-presenting cells that activate other types of immune cells such as T-cells and natural killer cells [228] [229]. Another similar strategy consists of the modality of calreticulin protein release, which is a powerful immunostimulatory protein generated as a response to induced apoptotic stress conditions [230]. Chauhan et al. [231] made chitosan-coated Fe_3_O_4_ MNPs and analyzed the MHT effect and anti-tumor response of an ectopic tumor model of C6 glioma cells in rats. They injected the MNPs on days 1 and 7, and, after that, they noticed complete tumor reduction on day 32. The applied AMF has a frequency of 335 kHz and a magnetic field strength of 14 kA/m. Real-time gene expression tests of pro-inflammatory cytokines proved that the *IL-6* activation process has an important effect on immunomodulation. Carter et al. [232] proved that only the application of MHT based on superparamagnetic MNPs can trigger the activation of immune system cells. After an in vivo experiment consisting of a GL261 murine glioma model and injection of Perimag-COOH MNPs directly into the tumor, cytotoxic T cells and increased production were activated in the animal lymph nodes. The authors concluded that MHT is a treatment that not only produces cancer cell death but also activates the animal’s immune system to fight against cancer, thus inducing long-term immunity. Regarding the heat shock protein effect, we have already presented a vital study [132] that underlines the positive cumulative effect of MHT and immunotherapy with potential clinical translation into GBM treatment. 

Other immunotherapy and MHT combined strategies based on cytokines were developed. These signaling molecules regulate the immune system and control inflammation by increasing the cytotoxic T lymphocyte effect. Anti-cancer therapies include the use of interferon (IFN), colony-stimulating factors, and tumor necrosis factors. Unfortunately, this single strategy based on cytokines proved to have limited positive effects in clinical trials due to the short half-life of cytokines and narrow therapeutic window [126]. Instead, combining cytokines with MHT [145] proved to be an efficient way to tackle GBM. 

**Figure 8:**
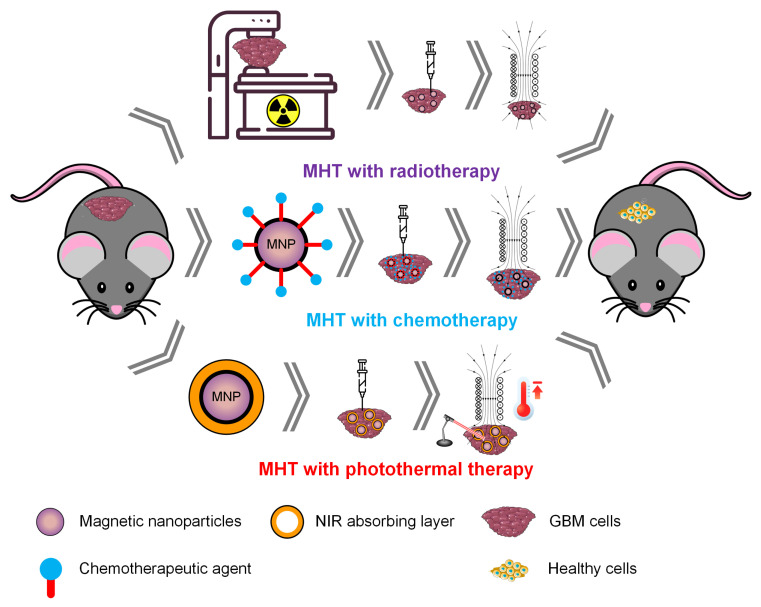
Combined strategies used in GBM treatment. This Figure was generated using images assembled from Servier Medical Art, which are licensed under a Creative Commons Attribution 3.0 unported license (https://smart.servier.com, accessed on 20 July 2024).

From the studies presented in this section, it is clear that MHT, in combination with other therapies, holds significant promise for curing GBM. We believe that in the near future, these combined strategies, especially those involving light-based therapy and immunotherapy, will be developed and adopted in clinical trials. These combinations not only allow for a decrease in MNP dose and a reduction in related toxicity associated with transition metal ions but also address multiple mechanisms based on different physical phenomena, which can work together to effectively eliminate cancer cells. Figure 8 shows some combined strategies used in GBM treatment.

**Table 5:** Relevant literature studies related to GBM treatment based on combinations between MHT and other therapies.

Combined Therapy Type	Implant/MNPs	AMF Conditions	Therapy Parameters	Study Type	Cell Line/Animal Characteristics/Humans	Remarks	Ref.
RT + MHT	Fe-Pt implant, 1.8 mm diameter, 15 ÷ 20 mm length	240 kHz/induction coil with a 30 cm diameter	Interstitial hyperthermia temperature between 44 ÷ 46 °C and 30 ÷ 60 min/2 or 3 per week combined with RT	In vivo	Human/7 cases of metastatic brain tumor	Interstitial magnetic hyperthermia combined with RT is an efficient way to treat intracranial metastases. Complete healing in 2 patients	[170]
	Silver nanoparticle (AgNP)-mediated RT with MHT based on γ–Fe_2_O_3_ MNPs	40 kHz/100 kA/m	Hyperthermia temperature of 42 °C for 15 min/Combined with 0 ÷ 6 Gy	In vitro	U251	Radio- and thermos-sensitivity on U251. The lowest cell survival rate was obtained under AMF application and ionizing radiation of 6 Gy	[171]
	Fe_3_O_4_ core of 12 nm diameter with aminosilane coating/Magnetic fluid MFL AS1 (NanoTherm^®^ AS1; MagForce Nanotechnologies) with 112 mg/mL MNPs concentration	100 kHz/2 ÷ 15 kA/m	Hyperthermia temperature of 43 °C/six semi-weekly sessions/1 h each thermotherapy session/Combined with 30 Gy biologically equivalent median dose administrated fractionated as 5 × 2 Gy per week	In vivo–Clinical trial	Human/66 patients (59 with recurrent GBM)	An important increase in the overall survival rate was noticed in the case of this combined therapy that uses a low radiotherapy dosage	[169]
Chemotherapy + MHT	Fe_3_O_4_ MNPs and 5-FU were encapsulated within chitosan nanoparticles	180 kHz/35 kA/m/10 kW induction heating system coupled with a 9-turn coil 5 cm in diameter	First MHT was applied for 20 min followed by 2 MHT sessions with a 1-day pause	In vitro	A-172	The combined nanoparticles were successfully internalized by A-172 cells, and, through a combination of the two treatments, cell apoptosis was obtained. Apoptosis was confirmed by densification of the cytoplasm, cell shrinkage, and tighter packing of cell organelles	[193]
	Magnetic core–shell MNP-mediated delivery of a mitochondria-targeting pro-apoptotic amphipathic tail-anchoring peptide (ATAP)	300 kHz/5 kA/m	A temperature of 43 °C was obtained after the MNPs-ATAP-treated cells were subjected to MHT for 45 min	In vitro	U87 MG	The MNPs-ATAP system in combination with MHT conducted to an important apoptotic effect related to induced mitochondrial dysfunction of cancer cells	[194]
	Magnetoliposomes with encapsulated doxorubicin	3.5 MHz/30 kA/m/applied 20 min	At a temperature of 43 °C, the encapsulated chemotherapeutic drug was released in a guided way	In vitro/In vivo	C6/Adult Sprague Dawley rat	The in vitro experiments demonstrated that the cell viability decreased to 79.2% for only MHT treatment, to 47.4% for only doxorubicin effect, and for a combination of the two strategies, it reached a value of 17.3%. Regarding the in vivo study, an enhanced effect of tumor volume growth inhibition followed by a full regression of the tumor was achieved	[150]
PTT + MHT+ Immunotherapy	Core–shell Fe_3_O_4_@Au MNPs combined with chemotherapeutic antibody Cetuximab (C225)	230 kHz/30 A/3 cycles of AMF	AMF cycles were combined with three irradiation sessions with NIR laser light (635 nm, 0.3 W/cm^2^)/30 min each/24 h pause	In vitro	U251	In comparison with the control group, tumor growth was inhibited in the case of the combined strategy. The high affinity of C225 towards cancer cell receptors generated increased cell uptake for MNPs	[205]
PTT + MHT	Citric-acid-coated iron oxide MNPs that were encapsulated in cationic liposomes containing 1,2-dipalmitoyl-sn-glycero-3-phosphocholine (DPPC), dimethyldioctadecyl ammonium bromide (DDAB), cholesterol (CH), cationic lipid dimethyldioctadecyl ammonium bromide (DDAB)	5 kHz/-	AMF session was combined with NIR laser action (808 nm, 1.8 W/cm^2^). A temperature of 56 °C was achieved	In vitro	U87	Cationic magnetoliposomes exhibited a promising effect in killing the cancer cells when PTT was combined with MHT	[206]
	Mn-doped magnetic nanoclusters	405 kHz/168 Oe/20 min	AMF session was combined with the effect of a near-infrared continuous laser (750 nm)	In vitro	C6	The combination between MHT and PTT generated an increased toxicity to cancer cells by ROS-mediated apoptosis. The SAR value of the Mn-doped nanocluster was about 600 W/g	[140]
PDT + MHT	Chloroaluminum-phthalocyanine (0.05 mg/mL) encapsulated-magnetic nanoemulsion	1 MHz/40 Oe	The MHT therapy was combined with PDT (670 nm wavelength, 700 mJ/cm^2^ energy density)	In vitro	U87 MG, T98G	Cell viability was found to decrease by 70% for this combined strategy, and only by 15% for MHT applied as single therapy	[212,213]
Immunotherapy + MHT	Fe_3_O_4_ liposomes in conjunction with HSP70	118 kHz/384 Oe	The MHT treatment was combined with immunotherapy based on HSP70	In vitro/In vivo	T-9/Fisher rat	Important tumor regression combined with enhanced tumor immunity was achieved	[132]
	Fe_3_O_4_ liposomes in conjunction with *TNF*–α gene therapy	118 kHz/384 Oe	The MHT treatment was combined with immunotherapy based on TNF-α	In vitro/In vivo	U251-SP/mice	The *TNF–*α property to inhibit neovascular apparition and to damage the GBM blood vessels combined with MHT led to efficient tumor volume reduction	[145]
	Fe_3_O_4_ MNPs combined with targeted heat shock protein 90 inhibition (HSP 90) (17-DMAG)	335 kHz/175 Oe	HSP90 was overexpressed for both cell lines compared to control samples under the MHT effect	In vitro	C6, U87-MG	Through the use of 17-DMAG, an HSP90 inhibition was noticed, and glioma cell sensitivity to MHT was increased	[219]

## 5. Clinical Studies of MHT in the Case of Patients with GBM 

As we have underlined in previous sections for MHT treatment application, it is undoubtedly necessary to have an AMF generator [233]. The solenoidal coil represents the main component of different developed systems. The target region is introduced inside the coil and placed under a uniform magnetic field. Today, commercially available coils are produced by MagForce Nanotechnologies AG, Berlin, Germany, and Nanoscale Biomagnetics, Zaragoza, Spain [46,141,143,234]. It can be estimated that the coils that were used for in vitro or in vivo studies can be modified to be applied in the case of humans, but, unfortunately, in almost all the cases, important problems consisting of the fact that field uniformity is limited to the central zone of the coils and asymmetric field distribution could occur through transverse planes are identified [235]. A coil that has planar turns, field concentrator pieces placed on coil ends, and wider leads was designed by Bordelon et al. [235] to produce a highly uniform magnetic field. The hyperthermia and thermoablation system called MFH 300F Nanoactivator^®^, MagForce Nanotechnologies AG, Berlin, Germany, has been approved in the European Union for the clinical application of MHT to different types of oncological diseases [169,236,237,238,239,240]. This device generates a uniform AMF with a frequency of 100 kHz and a magnetic field strength of 18 kA/m. In addition, the field applicator has a diameter of 20 cm and can accommodate different types and shapes of tumors. Dedicated software for treatment planning, NanoPlan^®^, MagForce Nanotechnologies, is involved for accurate temperature control and coupled with the AMF generator. At the European level, the quality assurance guidelines for superficial hyperthermia were established by the European Society for Hyperthermic Oncology (ESHO) [241,242] and provide clinical conditions to link hyperthermia parameters to a tumor’s geometrical shape and size. All clinical trials must respect these regulations.

We have identified several clinical studies focusing on MHT therapy in human glioma patients. The first studies in this direction were conducted by Stea et al. [243,244,245] and Iacono et al. [246], which investigated, in a phase I clinical trial, the effect of MHT used in conjunction with RT in the case of human subjects with primary or recurrent GBM. Although these investigations do not report MNP use, they must be mentioned, considering their practical importance. In these studies, 28 patients received wire implants manufactured from Ni-4% wt. Si, intratumorally inserted. A number of 11 patients received only one MHT session for 60 min, and, in the case of the remaining 17 patients, two AMF irradiations were applied. An overall survival time of about 15 months for patients with GBM was reported. Three significant complications were identified by the authors: secondary hydrocephalus due to catheter insertion, intracranial hemorrhage at wire implantation, and pneumocephalus generated by incorrect suture operation. Unfortunately, one patient died due to the increased volume of the Ni-Si implant. Focal seizures and cerebral edema were reported as minor complications that occurred in the case of 11 patients. After MHT sessions were applied, a temperature of 42 °C was obtained in the tumor region with temperature sensors placed mainly in the tumor core (60%), followed by tumor margins (35%), and healthy tissue localized in the tumor vicinity (3.5%). The main conclusion of the studies was that the combination of MHT and RT seems to be adequate for treating GBM patients, but important morbidities associated with this treatment route must be considered. A follow-up investigation [245] demonstrated that, in the case of patients with primary high-grade glioma tumors, the combination of MHT and RT generated an increase in the survival rate compared to RT used as a single therapy. On the other hand, no noticeable benefits were reported by comparing these two strategies for patients with recurrent high-grade glioma. It was concluded that for humans with metastases, it is advisable to apply only RT as a single therapy. Kobayashi et al. [247] developed a ferromagnetic implant (Fe-Pt) with a low Curie temperature adequate for MHT application. A number of 25 patients with brain tumors (glioblastoma and astrocytoma grade II) were treated for a maximum time of 23 weeks with a variable number of MHT sessions between 10 and 46. The repetition of MHT treatment in safe conditions was possible in the case of 23 patients, with an average response rate of about 35%. Implant migration and non-uniform thermal field distribution were reported as shortcomings. Side effects such as modification of cancerous cell morphology, thrombosis, or vascular problems were noticed in the places found near the area with coagulative necrosis in the implant vicinity. The authors concluded that no important other major drawbacks occurred.

**Figure 9:**
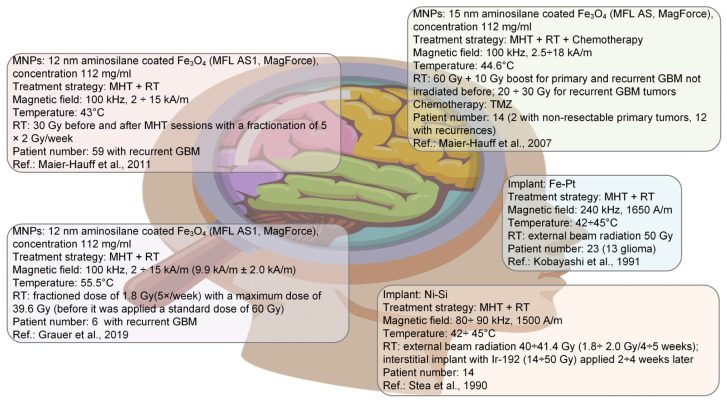
Main clinical trials performed on patients with GBM (Maier-Hauff et al., 2011 [169]; Grauer et al., 2019 [248]; Maier-Hauff et al., 2007 [236]; Kobayashi et al., 1991 [247]; Stea et al., 1990 [243]). This Figure was generated using an image from www.freepik.com, accessed on 21 July 2024.

Considering the analyses mentioned above performed based on ferromagnetic implants, different studies involving the use of MNPs for MHT applications were performed to observe the treatment’s most important shortcomings and side effects. Maier-Hauff et al. [236] performed a clinical study for twelve patients with recurrent GBM and two patients with primary GBM. They used a commercially magnetic fluid of aminosilane-coated iron oxide MNPs produced by MagForce Nanotechnologies, Germany. A high ferromagnetic concentration of 112 mg of Fe/mL was dispersed in water and injected into the tumor. They combined the MHT route with external beam radiotherapy. A standard AMF system MFH 300F (frequency of 100 kHz and variable magnetic field strength of 2.5 ÷ 18 kA/m) was used. An average temperature of 44.6 °C was achieved in the tumor zone. Patients received an average number of six MHT sessions and a single fraction of 2 Gy of a radiotherapy series with a median number of 30 Gy. MHT was well tolerated by all the patients with minor or absent side effects. It was concluded that the combination of MHT and RT is a good strategy for GBM treatment. The same research group [169] conducted an important large clinical trial that led, in 2012, to the European approval of MHT as adjuvant therapy in conjunction with RT for recurrent GBM. In this clinical trial, 59 patients with recurrent GBM were included. A median overall survival rate determined from the first diagnostic moment until the first recurrence apparition was established to be 13.5 months (95%CI: 17.2 ÷ 29.2). Magnetite MNPs were involved in the MHT strategy, which was combined with fractioned stereotactic radiotherapy with a median dose of about 30 Gy. Minor side effects such as headache, fever, tachycardia, moderate hypertension, and convulsion were reported. Only 14 patients reported a temporary worsening of their preexisting hemiparesis. The main drawback of this study was the necessity of removing all the metallic implants from the patient’s head. The main conclusion of this study was that a higher median survival rate can be achieved by applying MHT and RT combined therapy [169]. A recent and innovative investigation was reported by Grauer et al. [248] who combined MHT with RT once again. This study was performed on six patients with recurrent GBM. After tumor resection, the cavity wall was coated with 2 ÷3 layers of MNPs based on a hydroxycellulose mesh and fibrin glue, which was found to be adequate for increasing the stability of MNPs and providing adequate fixation. Patients received six semi-weekly AMF irradiation sessions for 1 h followed by RT in the case of four persons at a dose of 39.6 Gy. Of the four patients who were administered combined therapy, two patients exhibited no signs of tumor recurrence after 23 months. The median survival rate was estimated at 6.25 months, and the median overall survival rate was about 8.15 months. It was observed that after 2 ÷5 months, some clinical symptoms were present. CT scans revealed prominent edema placed around MNPs. Dexamethasone was administrated, and, in some cases, it was necessary to remove the MNPs surgically. Based on histopathology analyses, it was concluded that necrosis occurred in the zones near the MNPs, and it was also evidenced by an immune response with macrophage infiltration. The treatment was considered to exhibit positive effects on patients with recurrent GBM, although an important inflammatory reaction was observed in the vicinity of the resection cavity. Figure 9 synthesizes the main clinical trials found in the literature regarding the MHT treatment involved in GBM.

It can be noticed that MHT analysis is characterized by a lack of large phase III clinical trials that can explore the MHT effects in conjunction with chemotherapy and RT. Much more research is still necessary to establish a correct treatment route to increase the median survival rate.

## 6. Challenges and Future Perspectives

Our research has identified significant challenges in the field of MHT. The first is related to choosing the correct MNP ratio to be administered to achieve a moderate heating effect in the tumor zone. Another issue is linked to a proper understanding of the combining mechanisms, which occur in the case of MHT and other oncological approaches, to find adequate protocols to address different types of tumors and personalize the treatment for each patient’s anatomy. Many in vivo studies must be performed to establish a direct link between the magnitude of heat and the MNP ratio deposited in the GBM tumor and the value of MNP concentration needed in other body zones with metastases. 

These challenges must be addressed and solved as future perspectives in GBM treatment by applying the MHT protocol. First of all, regarding the correct dose in the metastases, a new administration procedure must be developed. An answer can come from combining immunotherapy and MHT through the adaptation of immune system cells to transport MNPs and introduce them easily and quickly into the tumor microenvironment. Another research direction could be the development of functionalized MNPs capable of enhancing the therapeutic effect, as well as increasing the MNPs’ action time inside the tumors. By involving chemotherapy and immunotherapy, a remote heating control correlated with drug release can be locally applied, avoiding the systemic adverse effects that can occur in the case of classical approaches. In the case of combination with RT, radioisotopes can be associated with MNPs, permitting, in this way, the elimination of external radiation sources. Although some studies have been conducted, much more research on in vivo animal models must be carried out to establish a treatment protocol.

An important future direction can be considered in MNP manufacturing, which has a different chemical composition from classical iron oxides, which can enhance ROS and metallic ion delivery directly into the tumor to obtain faster cancerous cell death. In addition, researchers in the field must consider the development of MNPs characterized by an optimal interaction process with cells and reduced toxicity that is safe and without significant side effects, permitting tumor evolution monitoring with MRI devices.

Another future perspective must focus on the diminishing MNP ratio and its interaction with fluids with different viscosities because the tumor environment is complex and presents modified corporal fluids that can greatly influence MNP movements and magnetic behavior.

Another important direction must address easy, feasible, and large-scale production methods of MNPs. Although the experiments proved a high SAR value for MNPs manufactured through non-hydrolytic methods, it is hard to implement such technologies at the industrial level. Hydrolytic and non-hydrolytic methods must consider the use of biocompatible substances and a green route of production with no harmful environmental effects.

Considering all the challenges and future perspectives presented in this section, MHT treatment for GBM must be further developed to search for improved solutions, and many more experimental studies are necessary.

## 7. Conclusions

Glioblastoma treatment can be performed based on magnetic hyperthermia induced by magnetic nanoparticles under the influence of an external alternative magnetic field. It can be foreseen that MHT and other therapy routes can be combined successfully to treat primary or recurrent tumors.

A few possibilities are associated with GBM treatment based on MHT that are approved for human trials. Many non-commercial MNPs developed in different laboratories have proven to be highly efficient in inducing the death of brain cancer cells. These MNPs, characterized by their good magnetic properties and increased SAR value in the biological limit conditions for the applied AMF, must be further included in clinical trials.

The development of new therapeutic strategies, which have to include other treatment directions such as immunotherapy and chemotherapy, with devoted attention to an adequate MNP ratio related to the tumor’s nature and shape, remote particle transport control, and local drug release when necessary, should be addressed.

It can be anticipated that many patients worldwide will shortly benefit from MHT therapy.

## Figures and Tables

**Table 1:** Magnetic and structural properties of pure IONs or combinations dedicated to MHT treatments.

MNP	*M*_s_ (emu/g)	*M*_r_/*M*_s_	*H*_c_ (Oe)	*K* (J/m^3^)	*D* (nm)	MNP Geometrical Attributes	Remarks	Ref.
Fe_3_O_4_	85 ÷ 110	-	-	1.3 ± (0.2) × 10^4^	9 ÷ 15	Spherical	All the magnetic properties were measured at 300 K	[51]
	60	0.4	170	-	5.7 ÷ 11	Large-diameter MNPs were a mixture of cubic, diamond, and triangular. Small-diameter MNPs were spherical	The hysteresis loops were measured at 5 K	[54]
	65 ÷ 92	-	70 ÷ 340	-	7.4 ÷ 45	Spherical	The hysteresis loops were measured at 5 K	[55]
	For 6.6 nm MNP, 80.8 at 5 K and 70.7 at 300 K; for 17.8 nm MNP, 91.3 at 5 K and 82.5 at 300 K	For 6.6 nm MNP, 0.28 at 5 K and 0.024 at 300 K; for 17.8 nm MNP, 0.29 at 5 K and 0.0076 at 300 K	For 6.6 nm MNP, 405.6 at 5 K and 14.7 at 300 K; for 17.8 nm MNP, 379.4 at 5 K and 3.4 at 300 K	4.74 × 10^5^ for 6.6 nm MNP, 1.11 × 10^5^ for 17.8 nm MNP	6.6 ÷ 17.8	Spherical	Magnetic properties were measured at 5 K and 300 K	[52]
	80 ÷ 90	-	141–500	-	12 ÷ 38	Cubic	SAR was between 400 and 800 W/g_Fe_	[56]
	78 ÷ 95	-	400–500 for MNPs with size below 20 nm; 250 ÷ 300 for larger particles	-	13 ÷ 40	Cubic	SAR was about 509 W/g_Fe_ at 320 kHz and 15 kA/m (*H* × *f* = 4.8 × 10^9^ kA/(ms))	[57]
	50 ÷ 81	0.03 ÷ 0.12	0 ÷ 103	(6.4 ÷ 9.5) × 10^3^	17 ÷ 47	Octopods	*M*_s_ and *H*_c_ were measured at 300 K	[58]
	20 ÷ 80	-	0–50	-	40 ÷ 70/5 ÷ 10	Nanorods	Magnetic measurements were performed at 300 K	[59]
	83 ÷ 88	0.38 ÷ 0.44	200 ÷ 290	(1.8 ÷ 2.6) × 10^4^	20 ÷ 28	Nanoflowers	SAR value of 1992 ± 34 W/g	[60]
	68 ÷ 92	0 ÷ 0.14	0 ÷ 118	-	13 ÷ 260	Octahedral	*M*_s_ was measured at 300 K. For 22 nm MNPs, SAR = (200 ÷ 800) W/g; for 43 nm MNPs, SAR = (250 ÷ 2400) W/g	
Fe_2_O_3_	2.8 × 10^5^/ 3.9 × 10^5^/ 3.9 × 10^5^	-	-	2.6 × 10^4^/ 8 × 10^2^/ 1.1 × 10^3^	10/ 18/ 22	Spheres/Cubes/Nanoflowers	τ_N_, τ_B_ (s): 2 × 10^−6^, 2.4 × 10^−8^/8 × 10^−6^, 3 × 10^−9^/2 × 10^−5^, 4.6 × 10^−9^. The LRT model was valid only for spheres	[61]
Au@Fe_3_O_4_	28 ÷ 92	0.29 ÷ 0.32	280 ÷ 550	6 × 10^3^	21 ÷ 52	Dimers	Gold shell provided improved biocompatibility	[62]
CoFe_2_O_4_@Fe_3_O_4_	108	-	2530	2 × 10^4^	15	Spherical/Core–shell	-	[51]
Fe_3_O_4_@CoFe_2_O_4_	105	-	11,600	1.8 × 10^4^	15	Spherical/Core–shell	For a frequency of 500 Hz and a field amplitude of 37.3 kA/m (*H* × *f*) = 18.7 × 10^9^ a SAR of 2795 W/g was reported	[51]
Fe_3_O_4_@FeO	40 ÷ 80	-	1200 ÷ 5754	(1 ÷ 1.3) × 10^4^	16 ÷ 23	Cubic/Core–shell	The magnetic measurements were performed at 10 K	[63,64]
FeO/Fe_3_O_4_	110	0.7	583	-	-	Clusters centrosymmetric	Magnetization measurements were performed at 300 K, and the coercivity was determined at 10 K	[65]
	82	0.7	520	-	-	Clusters dimers/trimers		

**Table 2:** Examples of MNPs synthetized trough colloidal methods and heating efficiency.

MNP	Synthesis Method/Producer	Particle Size (nm)/Shape	SAR (W/g)	*H* × *f* (A/(ms))	Remarks	Ref.
γ-Fe_2_O_3_	Co-precipitation Acid treatment	8 11 13 Spherical	10 40 58	3.92 × 10^9^	The synthesis method is adequate for a large amount of particles. Cheap, easy, and reproducible protocol. The optimal particle size correlated with SAR was established to be around 12 nm.	[85]
Commercial MNPs–Resovist^®^ (FDA approved)	Co-precipitation/ Bayer-Schering	20	26.8	1.86 × 10^9^	Compared to other commercial MNPs similar magnetic heating efficiency was observed between Micromod’s nanomag-D 100 nm, Resovist, and Chemicell’s aged fluidmag-D 50 nm.	[86]
Commercial MNPs–Feraheme^®^ (Ferumoxytol) (FDA approved) (γ-Fe_2_O_3_)	Co-precipitation/Berlex Laboratories	30	50.5	2.75 × 10^9^	FDA approval permitted the use of commercial Feraheme^®^ as an MHT nano heater. The particles coated with a polymer matrix (dextran) showed excellent heat transfer properties, being a good candidate for GBM treatments.	[87]
Fe_3_O_4_	Co-precipitation; the synthesis process was conducted in an automated batch reactor Atlas Potassium (Syrris)	13 18 20 Faceted	46.64 86.87 51.90	3.58 × 10^9^	Large quantities of MNPs were prepared. The highest SAR value was obtained for particles with a size of about 18 nm. All the particles were adequate for MHT.	[88]
Fe_3_O_4_	Oxidative precipitation	22 26 34 Cubic	130 170 120	2.54 × 10^9^	High-quality Fe_3_O_4_ nanocrystals were prepared. The highest SAR was noticed for a cubic particle with an edge of 170 nm.	[72]
Fe_3_O_4_ and ε-Fe_2_O_3_	Oxidative precipitation	22 Spherical (Fe_3_O_4_), Acicular (ε-Fe_2_O_3_)	95	2.49 × 10^9^	The particle mixture exhibited a higher SAR value. The MNPs are a good candidate for AMF cancer therapy.	[73]
Fe_3_O_4_ and γ-Fe_2_O_3_	Thermal decomposition	18/Octahedral 22/Truncated octahedral	124 320	3.06 × 10^9^	The particles exhibited superparamagnetic behavior at room temperature. The predominant magnetic relaxation phenomena consisted of Neel processes. Higher SAR values were obtained compared to other synthesis routes.	[89]
IONs	Thermal decomposition; binary solvent mixture approach	14 19 24 35 Cubic	360 620 650 300	4.80 × 10^9^	High SAR values were noticed, and it was concluded that the developed IONs could be successfully used as nano heaters.	[56]
Fe_3_O_4_ and/or γ-Fe_2_O_3_	Thermal decomposition	14 18 22 Faceted	70 80 95	3.06 × 10^9^	Superparamagnetic nanocrystalline MNPs with sizes higher than 10 nm were obtained. All SAR values were in biological limits.	[84]
Fe_3_O_4_	Thermal decomposition	5 10 14 13 Spherical	180 130 447 200	9.80 × 10^9^	A high SAR rate was noticed when the polydispersity of the magnetic fluid decreased. The 14 nm diameter particles exhibited the highest value of SAR.	[83]
γ-Fe_2_O_3_	Solvothermal/Polyol route	21 24 28 34 38 Nanoflowers	500 1992 1944 1230 787	4.40 × 10^9^ 1.51 × 10^9^–for all the other sizes	Nanoflowers beyond the superparamagnetic range were synthetized. The flowers comprised independent crystals with an average size of 11 nm. The polycrystalline character generated an increase in heating power, making these nanoparticles suitable for GBM treatment, even for recurrent tumors.	[60]
α-Fe_2_O_3_	Aerial oxidation and reduction	26 × 98 25 × 97 16 × 87	190 260 370	4.40 × 10^9^	To be used as MHT agents, the SAR values must be improved in the low-field domain.	[90]

**Table 3:** In vitro studies for MHT dedicated to GBM analysis.

MNPs/Quantity	Cell Lines	Particle Diameter/Shape	AMF Conditions–H (Oe), f (kHz)	SAR (W/g)	Temperature (°C)	Main Physical Phenomenon	Ref.
γ-Fe_2_O_3_ coated with polyol/50 μg/mL	HUVEC, U89-MG	10 nm/spherical	289.7 Oe/700 kHz	HUVEC: 114 W/g ± 21; U87-MG 178 W/g ± 37	42 °C	Cell thermospecificity	[129]
Zn_0.9_Fe_2.1_O_4_/50 μg/mL	HUVEC, U89-MG	11 nm/spherical	289.7 Oe/700 kHz	36 W/g	41.5 °C	MNPs magnetic property tunning based on Zn doping	[130]
MNPs coated with polyethyleneimine (PEI)/10 μg/mL ÷ 100 μg/mL	SH-SY5Y micro-tumor-phantoms	-	299.71 Oe/570 kHz	239 ± 19 W/g in water	46 °C	Comparison between MHT and hyperthermia mediated through exogenous heating	[131]
Fe_3_O_4_ cationic liposomes (TMAG, DLPC, DOPE (1:2:2)/100 μg/mL	T-9 cell pellets	35 nm	383.72 Oe/118 kHz	-	42 °C	MHT combined with an innovative vaccination therapy	[132]
Fe_3_O_4_ cationic liposomes (TMAG, DLPC, DOPE (1:2:2)/7.2 mg/mL	T-9	35 nm	384 Oe/118 kHz	-	43 °C	Cancer cells were targeted and intracellular heated magnetoliposomes	[133]
Fe_3_O_4_/PEGMMA-PEGDMA/7.9 mg/mL	M059K	20–30 nm	225.72 Oe/297 kHz	-	63 °C	An innovative magnetic gel suitable for both thermal ablation and magnetic hyperthermia as a function of the applied magnetic field strength	[134]
Fe_3_O_4_@γ-Fe_2_O_3_ nanoparticles coated based on polyphenol/100 μg/mL	BV-2	10–14 nm	300 Oe/570 kHz	For cinnamon–MNPs, 335.7 W/g_Fe3O4_; for synthetic vanilla MNPs, 78.9 W/g_Fe3O4_; and for vanilla pods, MNPs 234 W/g_Fe3O4_	46 °C	An eco-friendly synthesis route was developed for obtaining highly biocompatible MNPs	[135]
Magnetosomes coated with chitosan/1 mg/mL	GL-261	-	340 Oe/198 kHz	125 ± 5 W/g_Fe_ for chitosan coated, 120 ± 4.7 W/g_Fe_ for PEI coated, and 72 ± 2.8 W/g_Fe_ for neridronate-coated samples	43 °C	Magnetosomes with high biocompatibility and potential application in MHT treatment	[136]
STE-Fe_3_O_4_/100 μg/mL	C6	49.77 nm/spherical	168 Oe/405 kHz	73.18 W/g, 1867.01 W/g_Fe_	43 °C	The natural plant-based coating prevented MNP agglomeration, increased cell uptake, and prolonged the retention time	[137]
Coated magnetosomes/1 mg/mL	GL-261	40 nm/cubo-octahedral	34 mT ÷ 47 mT/198 kHz	89 W/g_Fe_ ÷ 196 W/g_Fe_	43 °C ÷ 46 °C	Magnetosomes isolated from magnetotactic bacteria with high biocompatibility	[138]
Mn-doped magnetic nanoclusters/250 μg/mL	C6	133.53 ± 10.46 nm	168 Oe/405 kHz	600 W/g, 2197.80 W/g_(Fe+Mn)_	-	The importance of a bimodal application of Mn-doped magnetic clusters in magneto-photo thermotherapy of GBM was evidenced	[140]

## Data Availability

Not applicable.
